# Smad3 promotes cancer progression by inhibiting E4BP4-mediated NK cell development

**DOI:** 10.1038/ncomms14677

**Published:** 2017-03-06

**Authors:** Patrick Ming-Kuen Tang, Shuang Zhou, Xiao-Ming Meng, Qing-Ming Wang, Chun-Jie Li, Guang-Yu Lian, Xiao-Ru Huang, Yong-Jiang Tang, Xin-Yuan Guan, Bryan Ping-Yen Yan, Ka-Fai To, Hui-Yao Lan

**Affiliations:** 1 Department of Anatomical and Cellular Pathology, The Chinese University of Hong Kong, Hong Kong SAR 999077, China; 2 Li Ka Shing Institute of Health Sciences and Department of Medicine & Therapeutics, The Chinese University of Hong Kong, Hong Kong SAR 999077, China; 3 Clinical Translational Research Center, Shanghai Pulmonary Hospital, Shanghai 200092, China; 4 Department of Histology and Embryology, Tongji University School of Medicine, Tongji University Cancer Institute, Shanghai 200092, China; 5 Department of Clinical Oncology, The University of Hong Kong, Hong Kong SAR 999077, China

## Abstract

TGF-β is known to influence tumour progression. Here we report an additional role of Smad3 in the tumour microenvironment regulating cancer progression. Deletion or inhibition of Smad3 in the tumour microenvironment suppresses tumour growth, invasion and metastasis in two syngeneic mouse tumour models. Smad3^−/−^ bone marrow gives rise to an expanded NK cell population with enhanced tumour-suppressive activities *in vivo*, and promotes differentiation of NK cells *ex vivo*. We identify E4BP4/NFIL3 as a direct Smad3 target gene critical for NK cell differentiation. Smad3 suppresses transcription of IFN-γ via E4BP4 in a T-bet independent manner. Therefore disruption of Smad3 enhances both the E4BP4-mediated NK cell differentiation and anti-cancer effector functions *in vivo* and *in vitro*. Furthermore, systemic treatment with a Smad3 inhibitor SIS3 effectively suppresses cancer progression. In summary, suppression of NK cell-mediated immunosurveillance via the Smad3-E4BP4 axis contributes to cancer progression. We propose targeting Smad3-dependent tumour microenvironment may represent an effective anti-cancer strategy.

## Abstract (web summary)

Smad3, a transcription factor activated by TGF-β, has been implicated in tumorigenesis. Here the authors show that Smad3 inhibits NK cell differentiation and effector function by repressing NFIL3, and that genetic or pharmacological blockade of Smad3 expands tumour-suppressive NK cells and restricts tumour growth in mice.

Cancer progression is dependent not only on the characteristics of cancer cells but also on the tumour microenvironment^1^,^2^. Transforming growth factor-β (TGF-β) has been suggested to play a suppressive role in carcinogenesis, but paradoxically cancer cells also produce TGF-β1 to support the tumour development^3^,^4^,^5^,^6^. Cancer cell-derived TGF-β can promote tumour growth by triggering angiogenesis, epithelial–mesenchymal transition and the matrix metalloproteinase (MMP) system for ECM degradation^3^,^4^,^5^,^6^. There is an increasing evidence that pro-tumorigenic microenvironment is promoted by TGF-β signalling^7^,^8^. Smad3 is a key mediator of canonical TGF-β signalling pathway and plays an important role in the TGF-β1-mediated transcriptional regulation^9^. Smad3-deficient mice exhibit impaired mucosal immunity and diminished T-cell responses to TGF-β^8^,^10^. The lack of Smad3 resulted in severe graft-versus host disease by promoting Th1 differentiation and granulocyte-mediated tissue injury^11^. Smad3 can bind to the Foxp3 enhancer for the development of regulatory T (Treg) cells and differentially regulates the induction of Treg and Th17 immune responses^12^,^13^. Thus mice lacking Smad3 have impaired Treg development^14^. TGF-β has a suppressive role in the cytolytic activity, especially on the interferon responsiveness of natural killer (NK) cells *in vitro*^15^,^16^. In addition, Trotta *et al*.^17^ elucidated that TGF-β/Smad3 signalling can suppress CD16-mediated interferon-gamma (IFN-γ) production in NK cells via reducing T-bet expression. Smad3 mutant mice were reported to develop metastatic colorectal cancer^18^, which may be related to chronic inflammation^19^,^20^. In contrast, mice lacking Smad3 were recently suggested to be resistant to chemical-induced skin carcinogenesis by Li *et al*.^21^. The discrepancy among the studies underlie the importance in further delineation of the mechanism of TGF-β/Smad signalling in tumour progression. Nevertheless, these studies suggested that Smad3 could be an important checkpoint for TGF-β signalling in the tumour microenvironment.

In the present study, we demonstrate the crucial role of Smad3-dependent tumour microenvironment in cancer progression by using two well-established syngeneic mouse tumour models. Mice lacking Smad3 are protected against tumour growth, invasion, metastasis and death. NK cell population in the Smad3^−/−^ tumour microenvironment is dramatically expanded. This observation led us to identify that E4BP4 (Nfil3), one of the master transcription factors for NK cell differentiation^22^,^23^,^24^, is a direct Smad3 target gene. Thus TGF-β/Smad3 signalling facilitates cancer progression by suppressing NK cell production via downregulating E4BP4. The crucial role of Smad3 is further supported by animal experiments with reconstitution of GFP^+^ Smad3^−/−^ bone marrow on Smad3^+/+^ mice and adoptive transfer of Smad3^−/−^ NK1.1^+^ cells into B16F10 tumour-bearing NOD/SCID mice. Furthermore, systemic treatment with a Smad3 inhibitor can suppress cancer progression by reversing the tumour promoting to an anticancer microenvironment *in vivo*. Therefore, our findings reveal the unexplored role of Smad3 in NK cell immunity and tumour microenvironment. The therapeutic potential of Smad3-targeted therapy for cancer treatment is also implicated.

## Results

### Smad3 is essential for cancer progression in mice

**Figure 1:**
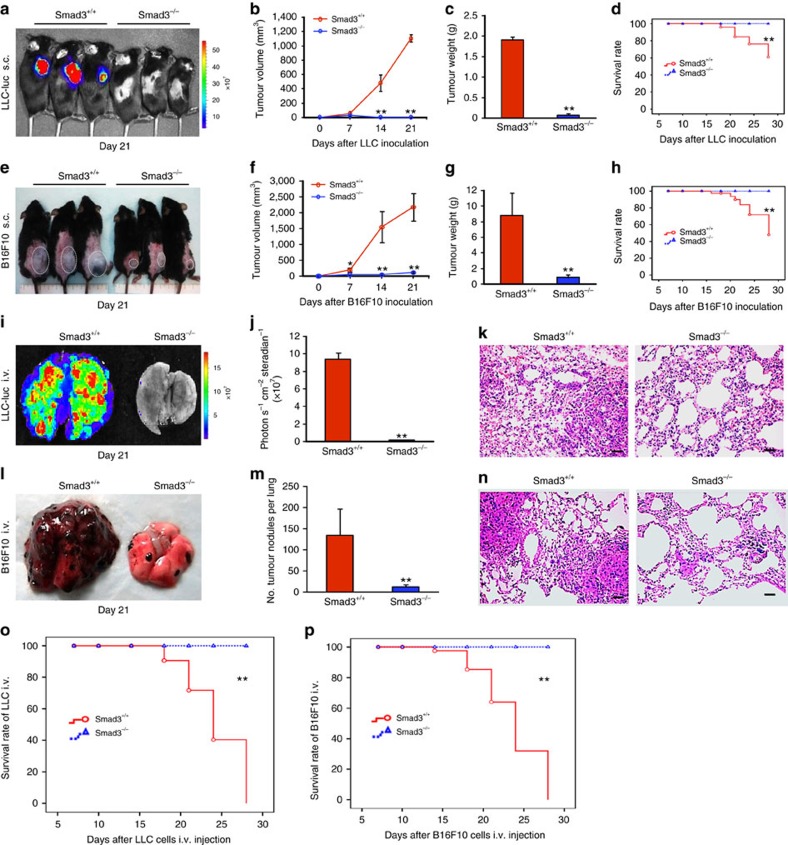
Mice lacking Smad3 are protected from tumour progression. LLC-luc cancer cells were s.c. inoculated into Smad3^+/+^ and Smad3^−/−^ mice (*n*=3) and examined by bioluminescence imaging on day 21 (**a**), tumour volumes (**b**), tumour weights on day 21 (**c**) and the Kaplan–Meier plot of survival (**d**). B16F10 cells (2 × 10^6^ cells per mouse) were s.c. inoculated into Smad3^+/+^ and Smad3^−/−^ mice (*n*=5) and examined for the growth patterns (highlighted with white circles) on day 21 (**e**), tumour volumes (**f**), tumour weights on day 21 (**g**) and the Kaplan–Meier plot of survival (**h**). LLC-luc cells (3 × 10^5^ cells per mouse) were i.v. inoculated into Smad3^+/+^ and Smad3^−/−^ mice (*n*=5) and examined for the metastatic growth patterns in the lung by bioluminescence imaging (**i**), quantitative photon intensities (**j**) and histology on H&E-stained sections (**k**). B16F10 cells (3 × 10^5^ cells per mouse) were i.v. inoculated in Smad3^+/+^ and Smad3^−/−^ mice (*n*=5) and examined for metastatic growth in the lung by macroscopic morphology (**l**), quantitative analysis of metastatic nodules (**m**) and histology on H&E-stained sections (**n**). Note that the metastatic B16F10 tumours in the lung are shown by the dark-black nodules grossly and the metastatic LLC or B16F10 nodules on H&E-stained sections are indicated with the white star (*). Kaplan–Meier plot of survival rates of metastatic LLC-luc (**o**) and B16F10 (**p**) tumour-bearing mice. Data represent the mean±s.d. for groups of mice as indicated in individual experiments above. *P<0.05, **P<0.01 compared with tumour-bearing Smad3^+/+^ mice analysed by analysis of variance. Scale bars, 50 μm.

As TGF-β/Smad signalling has been shown to be a pro-tumorigenic factor in cutaneous melanoma and lung cancer^25^,^26^, the role of Smad3 in tumour microenvironment was investigated by using two syngeneic mouse tumour models with luciferase-expressing invasive mouse lung carcinoma (LLC-luc) or melanoma (B16F10-luc) established in Smad3^−/−^ (deletion of exon 8 and disruption of exon 7^10^) and their littermate Smad3^+/+^ mice (C57BL/6J background). During the first week after subcutaneous inoculation of LLC-luc cells, we observed almost equal tumour growth in both Smad3^−/−^ and Smad3^+/+^ mice. However, in the subsequent weeks, significant tumour regression was found in Smad3^−/−^ mice where the tumours became undetectable by macroscopic and microscopic examinations and by bioluminescence imaging at week 3 (Fig. 1a–d). In contrast, the tumours in the Smad3^+/+^ mice grew rapidly and invasively, resulting in 40% mortality at week 3 (Fig. 1a–d). A similar pattern of cancer progression was observed in Smad3^−/−^ and Smad3^+/+^ mice bearing a highly invasive melanoma cancer cell line B16F10 (Fig. 1e–g). In the B16F10 tumour-bearing Smad3^+/+^ mice, melanoma grew rapidly and invasively with metastasis to the lymph nodes, lung and colon, resulting in 60% mortality at week 3 (Fig. 1h and Supplementary Fig. 1). In contrast, these malignant features were absent in the tumour-bearing Smad3^−/−^ mice in which survival rate was 100% without features of metastases (Fig. 1h and Supplementary Fig. 1). In addition, the protective effect of Smad3^−/−^ microenvironment was further confirmed on a tumour rechallenging model (Supplementary Fig. 2A,B). These observations supported the importance of Smad3 in the tumour microenvironment for promoting tumour growth, invasion and metastasis. The supportive role of Smad3-dependent microenvironment in cancer metastasis was further examined in mouse metastatic models by intravenously (i.v.) administering LLC-luc or B16F10-luc cells into the mice. As shown in Fig. 1i–n, mice lacking Smad3 were resistant to cancer metastasis with barely detectable LLC-luc cancer cells and a few melanoma nodules in the lung. By contrast, Smad3^+/+^ mice developed massive lung cancer with a 100% mortality rate (Fig. 1o,p). These findings provided a clear and direct evidence for a crucial role of Smad3-dependent microenvironment in cancer progression.

### Smad3 suppresses NK cell immunity in tumour-bearing mice

**Figure 2:**
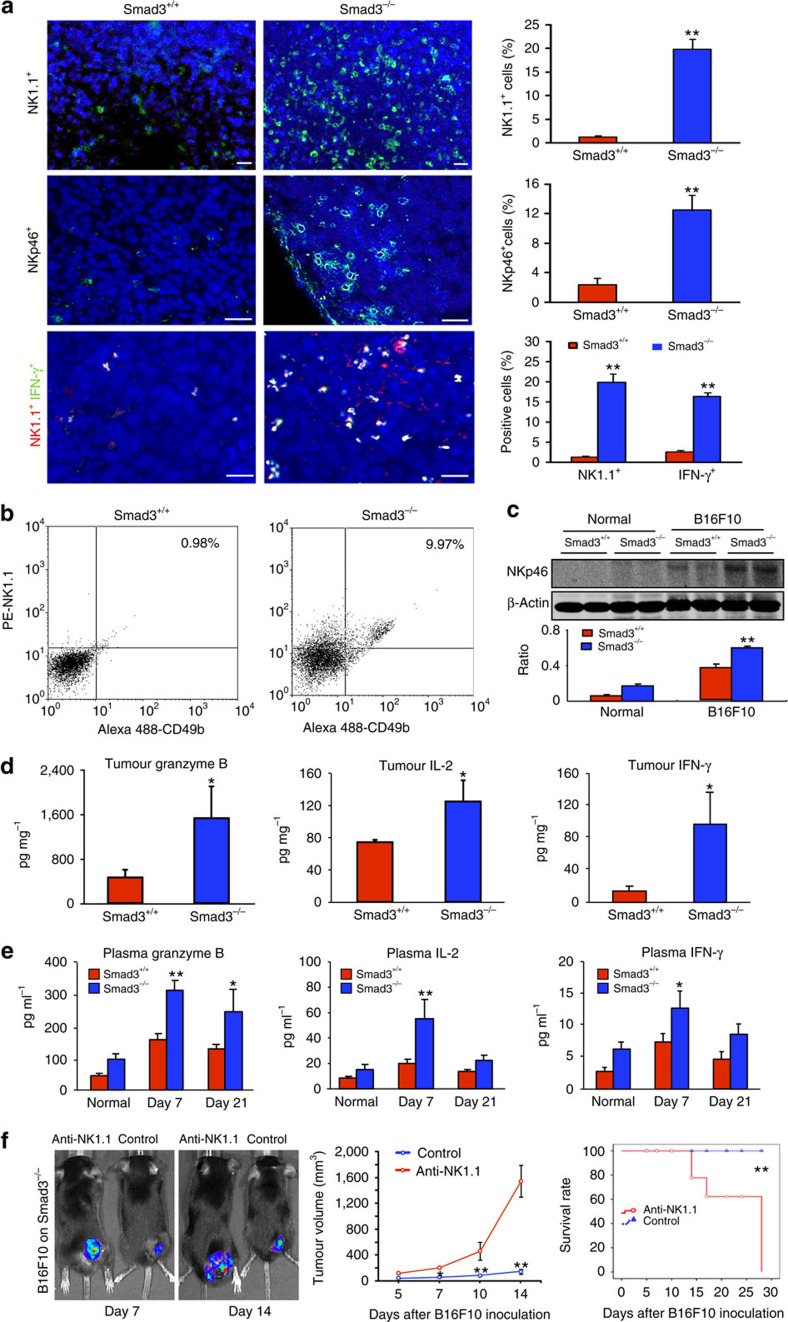
Smad3 facilitates cancer progression by suppressing host NK cell immunity in the tumour microenvironment. (**a**) Immunofluorescence detects the tumour-infiltrating NK1.1^+^, NKp46^+^ and NK1.1^+^ INF-γ^+^ NK cells in the B16F10 tumour collected on day 7. Representative images of tumour sections stained with the antibodies recognizing NK1.1 (green, upper panel), NKp46 (green, middle panel), NK1.1 (red) and IFN-γ (green, lower panel) are shown. Nuclei were counterstained with DAPI (blue), and the percentage of positive cells in the tumour tissues of Smad3^−/−^ or Smad3^+/+^ mice are shown (right panel). (**b**) Two-colour flow cytometry shows the population of tumour-infiltrating NK1.1^+^ CD49b^+^ cells in the B16F10 tumour. (**c**) Western blotting analysis detects the NKp46 expression within the tumour tissues. (**d**,**e**) Enzyme-linked immunosorbent assay analysis determines the levels of granzyme B, IL-2 and IFN-γ in the tumour tissues (**d**) and circulation (**e**). (**f**) Effects of NK cell depletion on cancer progression in B16F10 tumour-bearing Smad3^−/−^ mice as determined by bioluminescent imaging, tumour volume measurement and the survival rate. Data represent mean±s.d. for groups of 3–5 mice. *P<0.05, **P<0.01 compared with B16F10 tumour-bearing Smad3^+/+^ (**a**–**e**) or Smad3^−/−^ mice (**f**) analysed by analysis of variance. Scale bars, 50 (upper panel in **a**) and 100 μm.

We next investigated the underlying mechanisms whereby mice lacking Smad3 were protected against cancer growth and metastasis. A 10-fold increase in the number of tumour-infiltrating NK1.1^+^ NKp46^+^ and NK1.1^+^ IFN-γ^+^ NK cells was found in the B16F10 tumour-bearing mice lacking Smad3 compared with the Smad3^+/+^ group (Fig. 2a–c). The inhibitory effect of Smad3-dependent microenvironment on NK cell population was further investigated. Native Smad3^−/−^ mice (without tumour inoculation) showed slight increase in mature NK1.1^+^ CD49b^+^ NK cells in the bone marrow, spleen, lung and peripheral blood compared with Smad3^+/+^ mice (Supplementary Fig. 3A). The population of NK1.1^+^ CD49b^+^ NK cells was further increased after cancer inoculation, resulting in a 10-fold increase in NK1.1^+^ CD49b^+^ NK cells in the peripheral blood and lung and a 3-fold increase in the spleen compared with the tumour-bearing Smad3^+/+^ mice (Supplementary Fig.3B,C). The increase in mature NK1.1^+^ NKp46^+^ NK cells was also observed in the tumour, spleen and blood of the B16F10-rechallenged mice lacking Smad3 (Supplementary Fig. 2C,D). In addition, deletion of Smad3 also enhanced the anticancer activities of NK cells by increasing the levels of granzyme B, interleukin (IL)-2 and IFN-γ locally within the cancer microenvironment and in the systemic circulation (Fig. 2d,e). The splenic NK cells isolated from the B16F10 tumour-bearing Smad3^−/−^ mice also exhibited a significant increase in the *ex vivo* cytotoxicity against tumour cells with higher levels of INF-γ production when compared with the NK cells obtained from the tumour-bearing Smad3^+/+^ mice (Supplementary Fig. 4). Moreover, a marked reduction in vascular endothelial growth factor (VEGF) expression, CD31^+^ blood vessels, CD4^+^ Foxp3^+^ Treg cells and the expression of MMP-2, MMP-9, MMP-13 and C-X-C motif chemokine receptor 4 (CXCR4) in the tumour stroma were observed in the Smad3^−/−^ tumour microenvironment (Supplementary Figs 5 and 6). In contrast, depletion of NK cells from the tumour-bearing hosts with a neutralizing antibody restored rapid progression of the B16F10 tumour only in Smad3^−/−^ mice but not in Smad3^+/+^ mice *in vivo* (Fig. 2f and Supplementary Fig. 7). These findings suggested an inhibitory role of Smad3 in NK cell development on a systemic level and a crucial role of NK cells in the Smad3-dependent tumour microenvironment.

### Smad3 tumour microenvironment is derived from bone marrow

**Figure 3:**
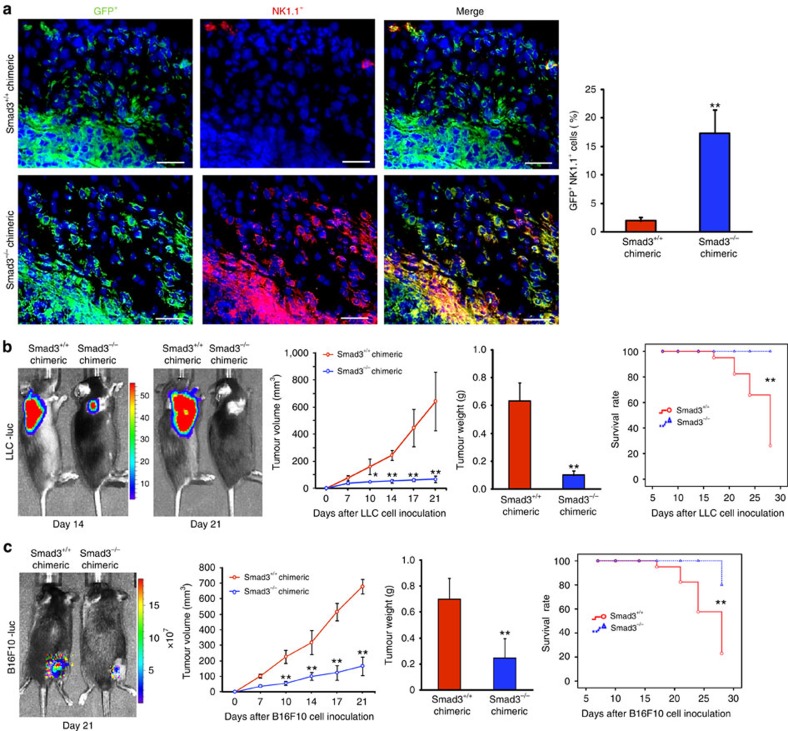
Smad3-dependent tumour microenvironment is derived from the bone marrow. LLC-luc or B16F10-luc cancer cells were s.c. inoculated into GFP^+^ Smad3^+/+^ and GFP^+^ Smad3^−/−^ chimeric mice and examined for: (**a**) bone marrow-derived NK cells (GFP^+^ NK1.1^+^) in the tumour tissues by two-colour immunofluorescence; (**b**,**c**) tumour growth patterns by bioluminescent imaging, tumour volumes, tumour weights on day 21 and the survival rate of LLC (**b**) or B16F10 (**c**) tumour-bearing chimeric mice. Data represent mean±s.d. for groups of 4–5 mice. *P<0.05, **P<0.01 compared with tumour-bearing Smad3^+/+^ chimeric mice received GFP^+^ Smad3^−/−^ bone marrow transplantation analysed by analysis of variance. Scale bars, 100 μm.

We then investigated the origin of Smad3-dependent tumour microenvironment by using GFP^+^ Smad3^−/−^ and GFP^+^ Smad3^+/+^ bone marrow chimeric mice bearing subcutaneous LLC-luc or B16F10-luc tumours. Our data showed that almost all stromal cells within the tumour tissue were derived from the transplanted bone marrow as recognized by their green fluorescent protein (GFP) expression (Fig. 3a). Compared with GFP^+^ Smad3^+/+^ chimeric mice, mice with GFP^+^ Smad3^−/−^ bone marrow exhibited significant reduction in the tumour growth and mortality rate (Fig. 3b,c), which was again associated with a 10-fold increase in GFP^+^ NK1.1^+^ cells in the tumour microenvironment (Fig. 3a). These results suggested that Smad3 may suppress host anticancer immunity at the bone marrow compartment. The inhibitory role of Smad3 on bone marrow-derived NK cell immunity was confirmed *in vitro*; the cancer-killing activity of NK cells differentiated from Smad3^−/−^ bone marrow cells was significantly higher than the Smad3^+/+^ control (Supplementary Fig. 8A). Interestingly, disruption of Smad3 (knockout or inhibition) facilitated NK cell maturation (NKp46^+^ IFN-γ^+^ cells) even in the presence of TGF-β1(Supplementary Fig. 8C). Therefore, deletion of Smad3 may enhance NK cell development in bone marrow and augmentation of host anticancer immunity at the distant tumour microenvironment.

### Smad3 suppresses E4BP4-dependent NK cell maturation

**Figure 4:**
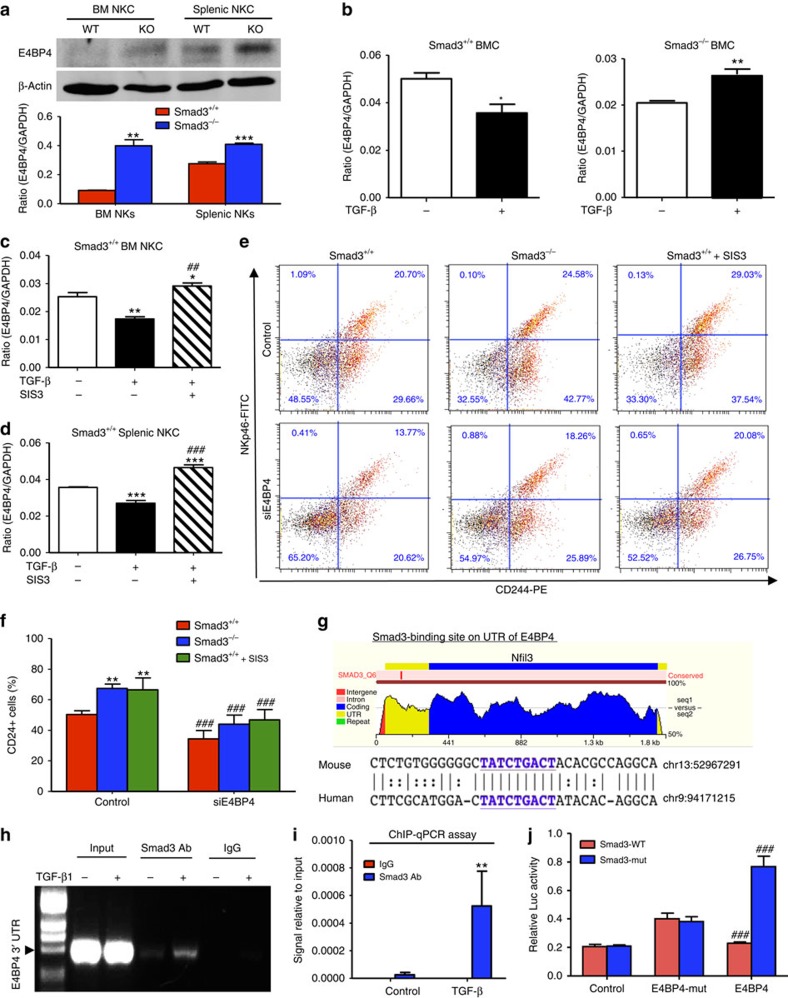
Smad3 suppresses NK cell differentiation by downregulating E4BP4 *in vivo* and *in vitro*. (**a**) Western blotting analysis shows the expression of E4BP4 in the NK cells isolated from the bone marrow and spleen of the B16F10 tumour-bearing Smad3^+/+^ and Smad3^−/−^ mice on day 10 after tumour inoculation. (**b**) Real-time PCR detects the effect of TGF-β1 (1 ng ml^−1^) on the transcription of E4BP4 in Smad3^+/+^ and Smad3^−/−^ bone marrow cells at 3 h. (**c**,**d**) Effect of SIS3 (2 μM) on TGF-β1-mediated E4BP4 mRNA expression in bone marrow or spleen-derived NK cells. (**e**) Two-color flow cytometry detects the effects of knockdown of E4BP4 and deletion (Smad3^−/−^) or inhibition (SIS3) of Smad3 in the production of bone-marrow cell derived CD244^+^ NKp46^−^ (NK progenitor) and CD244^+^ NKp46^+^ (immature NK) cells (upper panels) on Day 6. (**f**) Quantitative results of CD244^+^ cells from flow cytometry analysis. (**g**) A predicted SBS at the 3′ UTR of the evolutionarily conserved region of E4BP4 in human and mouse genomes (upper panel), indicated by bold and underlined (lower panel). (**h**,**i**) ChIP assay was performed with Smad3^+/+^ bone marrow cells stimulated with TGF-β1(1 ng ml^−1^) and the enrichment of Smad3 binding to the 3′ UTR of E4BP4 gene was qualified by PCR (**h**) and quantified by real-time PCR (**i**). (**j**) Dual luciferase assay shows the inhibitory effect of Smad3 binding on E4BP4 3′ UTR reporter activity. Data represent mean±s.e.m. for three independent experiments, *P<0.05, **P<0.01, ***P<0.001 compared with the control group (**b**–**d**,**i**) or Smad3^+/+^ cells (**a**,**f**); ^#^*P*<0.05, ^##^*P*<0.01, ^###^*P*<0.001 compared with the TGF-β1-treated group (**c**,**d**), individual controls (**f**) or E4BP4-mutant (**j**) analysed by analysis of variance.

As Smad3^−/−^ microenvironment was able to protect mice against cancer progression by dramatically increasing NK cell production (Figs 1, 2, 3 and Supplementary Figs 1 and 2), we further investigate the unexplored role of Smad3 as a negative regulator in the NK cell development. Western blottings showed that deletion of Smad3 increased the expression of E4BP4 (or Nfil3), an important transcription factor for NK lineage commitment^22^,^23^,^24^, in both bone marrow-derived and splenic NK cells from the Smad3^−/−^ mice on Day 10 after tumour inoculation (Fig. 4a). It suggested a close link between Smad3 and NK cell development in the tumour microenvironment. To examine the inhibitory role of TGF-β/Smad3 signalling in E4BP4, total bone marrow cells or untouched splenic NK cells from Smad3^+/+^ or Smad3^−/−^ mice were stimulated with TGF-β1 for 3 h and subjected for real-time PCR analysis. As shown in Fig. 4b–d, TGF-β1 significantly suppressed the transcription of E4BP4 mRNA in Smad3^+/+^ bone marrow cells and splenic NK cells, which was blunted when Smad3 was genetically deleted or pharmacologically inhibited with Smad3 inhibitor (SIS3). We next investigated the functional importance of E4BP4 in Smad3-mediated suppression of NK cell development by transfecting bone marrow cells from Smad3^+/+^ and Smad3^−/−^ mice with nonsense (control) or short interfering RNA (siRNA) against mouse E4BP4 (siE4BP4) and cultured under NK cell differentiation medium for 6 days. Real-time PCR detected that SIS3 treatment increased E4BP4 mRNA expression in Smad3^+/+^ NK cells, whereas transient knockdown with siE4BP4 resulted in significant inhibition of E4BP4 mRNA expression in bone marrow-derived Smad3^+/+^ and Smad3^−/−^ NK cells as well as SIS3-treated Smad3^+/+^ NK cells (Supplementary Fig. 9). Strikingly, while the production of CD244^+^ NKp46^+^ (immature NK cells) and CD244^+^ NKp46^−^ (NK cell progenitors) cells was significantly accelerated in Smad3^−/−^ or SIS3-treated Smad3^+/+^ bone marrow cells, this promoting effect was diminished when E4BP4 was knocked down (Fig. 4e,f), demonstrating the functional importance of E4BP4 in the Smad3-mediated NK cell differentiation. Furthermore, a conversed Smad3-binding site (SBS) was found within the 3′ UTR (untranslated region) of E4BP4 gene (Fig. 4g) by using ECR browser (rVista 2.0, http://rvista.dcode.org/)^27^. The physical binding of Smad3 on the UTR of E4BP4 gene was enriched by TGF-β1 stimulation as shown by the results of chromatin immunoprecipitation (ChIP) assay (Fig. 4h,i). Thus Smad3 transcriptionally inhibited E4BP4 in response to TGF-β1, which was reversed when Smad3 is mutated as demonstrated by the luciferase reporter assay (Fig. 4j). These findings revealed the inhibitory role of Smad3 in NK cell development via downregulating the transcription of its direct target gene E4BP4. Given that deficiency of Smad3 prevents TGF-β1-mediated inhibition of E4BP4 expression, targeting the TGF-β1/Smad3–E4BP4 axis to accelerate the development of NK cells may enhance their anticancer immunity within the tumour microenvironment.

### TGF-β1 suppresses NK cell immunity via Smad3–E4BP4 axis

**Figure 5:**
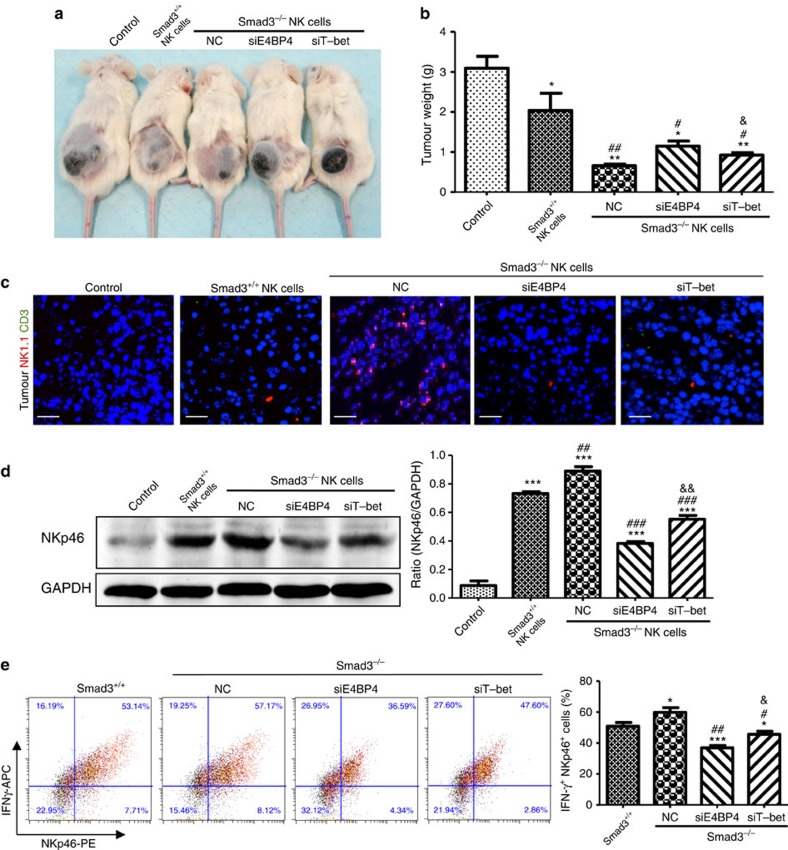
The anticancer effect of Smad3^−/−^ NK cells is dependent on E4BP4 more than on T-bet. (**a**) Saline (Control), nonsense-treated Smad3^+/+^ (Smad3^+/+^NK), nonsense-treated Smad3^−/−^ (NC) NK cells or Smad3^−/−^ NK cells with E4BP4 knockdown (siE4BP4) or T-bet knockdown (siT-bet) were infused (i.v.) into B16F10 tumour-bearing NOD/SCID mice and the antitumour effects are qualified by imaging on day 10 after NK cell infusion. (**b**) Tumour weight after NK cell infusion on day 10. (**c**) Tumour-associated NK cells detected by two-colour immunofluorescence with the anti-NK1.1 and anti-CD3 antibodies (scale bars, 100 μm). Note that most of anti-NK1.1^+^ cells within the tumour microenvironment are negative for CD3. (**d**,**e**) Effect of E4BP4 and T-bet on *ex vivo* NK differentiation in Smad3^+/+^ or Smad3^−/−^ bone marrow cells on day 7. Bone marrow cells were transfected with siE4BP4 or siT-bet and the NK cell population in B16F10 tumour was detected by western blotting with NKp46 (**d**) or by two-colour flow cytometry with the anti-NKp46 and IFN-γ antibodies (**e**). Data represent mean±s.e.m. for groups of three mice or at least three independent experiments. *P<0.05, **P<0.01, ***P<0.001 compared with the control group (**b**,**d**) or Smad3^+/+^ NK cells (**e**); ^#^*P*<0.05, ^##^*P*<0.01, ^###^*P*<0.001 compared with the S3WT-NK group; &*P*<0.05 and &&*P*<0.01 compared with the siE4BP4 group analysed by analysis of variance.

The specific role of Smad3 on NK cell-mediated antitumour activities was further examined by adoptive transfer of Smad3^+/+^ or Smad3^−/−^ NK1.1^+^ cells into the B16F10 tumour-bearing NOD/SCID mice in which the NK cell population was deficient^28^. Noticeably, NOD/SCID mice that received Smad3^−/−^ NK cells showed a greater inhibition of tumour growth compared with those that received Smad3^+/+^ NK cells (Fig. 5a,b). Trotta *et al*.^17^ recently reported of suppressed CD16-mediated IFN-γ production of NK cells by TGF-β1 via Smad3–T-bet-dependent pathway, so we compared the relative impact of E4BP4 versus T-bet on Smad3-mediated NK cell antitumour activities by adoptive transfer of the Smad3^−/−^ NK cells with or without knockdown of E4BP4 (Nfil3) or T-bet into the B16F10 tumour-bearing NOD/SCID mice. Interestingly, mice that received E4BP4 knockdown Smad3^−/−^ NK cells (siE4BP4) exhibited a higher tumour growth rate, which was associated with lower levels of infiltrating NK1.1^+^CD3^−^ NK cells and the expression of NKp46 when compared with those that received siT-bet Smad3^−/−^ NK cells (Fig. 5c,d). *In vitro* study also confirmed this observation that NK differentiation and IFN-γ expression were more significantly inhibited by knockdown of E4BP4 compared with that in T-bet knockdown Smad3^−/−^ NK cells (Fig. 5e). A direct E4BP4-binding site on the promoter of IFN-γ (which is 208 nt apart from the T-bet-binding site) is predicted by ECR browser and therefore the results supporting that knockdown of E4BP4 suppressed IFN-γ expression in a T-bet-independent manner (Supplementary Fig. 10).

### Targeting Smad3 protects against cancer progression

**Figure 6:**
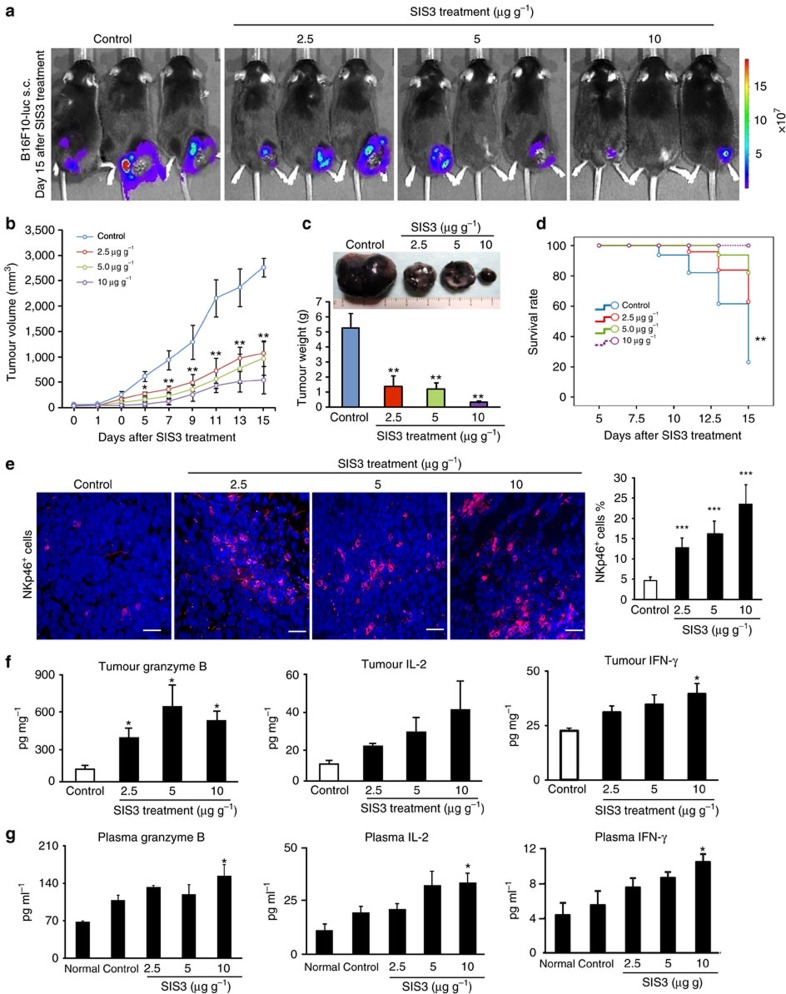
Inhibition of Smad3 prevents cancer progression by restoring NK cell anticancer immunity in tumour-bearing Smad3^+/+^ mice. B16F10-luc cancer cells were s.c. inoculated into Smad3^+/+^ mice and followed by treatment with various dosages of SIS3 (0, 2.5, 5.0 or 10 μg g^−1^ day^−1^, i.p.). (**a**) Representative bioluminescent images of the B16F10-luc tumours, (**b**) tumour volumes, (**c**) tumour weights on day 15 after treatment, (**d**) survival rates and (**e**) representative immunofluorescent images of tumour sections stained with the anti-NKp46 antibody (red) on day 15 after treatment. Nuclei were counterstained with DAPI (blue). (**f**,**g**) Enzyme-linked immunosorbent assay detects the levels of granzyme B, IL-2 and IFN-γ in the tumour tissues (**f**) and circulation (**g**) on day 15 after SIS3 treatment. Data represent mean±s.d. for groups of 4–6 mice. *P<0.05, **P<0.01, ***P<0.001 compared with control (saline-treated) analysed by analysis of variance. Scale bars, 100 μm.

**Figure 7:**
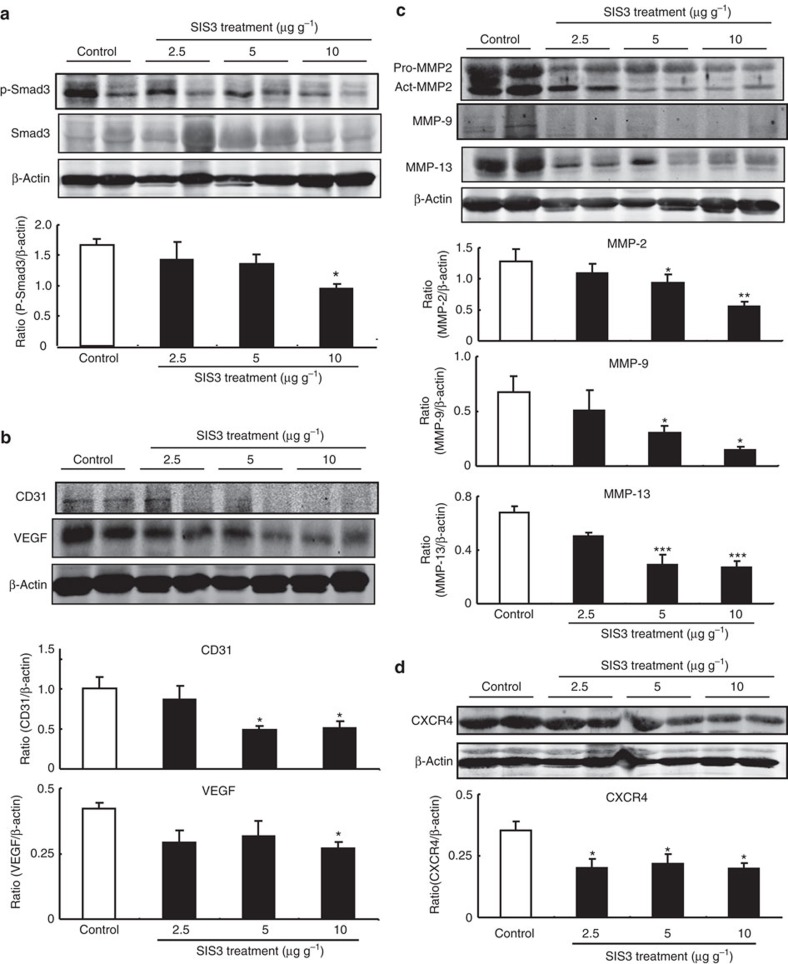
SIS3 treatment suppresses angiogenesis and the expression of tumour-invasive factors in B16F10 tumour-bearing Smad3^+/+^ mice. Western blotting analysis shows the intratumoural phosphorylation of Smad3 (**a**), angiogenesis including the expression of VEGF and CD31 proteins (**b**), pro- and active MMP-2, MMP-9 and MMP-13 expression (**c**) and CXCR4 expression (**d**) of the SIS3-treated mice. Data represent mean±s.e.m. for groups of 4–6 mice. *P<0.05, **P<0.01, ***P<0.001 compared with the control-treated group analysed by analysis of variance.

The encouraging findings from tumour-bearing Smad3^−/−^ mice leads us to further test a hypothesis that targeting Smad3-dependent tumour microenvironment may protect mice against cancer progression. This was examined on Smad3^+/+^ mice bearing B16F10 or LLC tumours by inactivating Smad3 signalling with an inhibitor SIS3 that specifically blocks the phosphorylation and DNA binding of Smad3 proteins^29^. The systemic treatment of SIS3 significantly inhibited the phosphorylation of Smad3 in both LLC and B16F10 tumour tissues and suppressed cancer progression in a dosage-dependent manner, resulting in a 100% survival rate (Figs 6a–d and 7a and Supplementary Figs 11A–D and 12A). More importantly, SIS3 treatment significantly increased NK cell production in a dose-dependent manner, showing up to a fivefold increase in NKp46^+^ cells in tumour tissues (Fig. 6e). Inhibition of Smad3 also enhanced anticancer activities of NK cells by increasing releases of granzyme B, IL-2 and IFN-γ locally within the tumour tissues and systemically in the circulation (Fig. 6f,g). Depletion of NK cells from SIS3-treated B16F10 tumour-bearing mice partially reversed the antitumour effects of SIS3 (Supplementary Fig. 13), which further supports the promoting role of Smad3-mediated NK suppression in cancer progression. *In vitro* study also confirmed this finding that pharmacological inhibition of Smad3 signalling with a SIS3 was capable of enhancing cancer-killing activities in both bone marrow-derived or splenic NK cells (Supplementary Fig. 8A,B). We demonstrated that the enhanced NK cell-mediated anticancer immunity has an important role in the anticancer effects of Smad3-dependent tumour microenvironment targeted treatment. Furthermore, systemic treatment of SIS3 also significantly altered the tumour-friendly microenvironment, including suppression on angiogenesis (VEGF expression and CD31^+^ vessels) and tumour-invasive factors (MMP-2, MMP-9, MMP-13 and CXCR4) (Fig. 7b–d, Supplementary Fig. 12B–D). *In vitro* treatment with SIS3 was also able to inhibit the proliferation of B16F10 melanoma cells in a dose-dependent manner (Supplementary Fig. 14) and this may also suggest a direct inhibitory effect of SIS3 on tumour cell growth. Taken together, our results revealed that targeting Smad3-dependent microenvironment may represent a novel and effective therapy for invasive cancer.

## Discussion

TGF-β has a supportive role in progression of established tumour and Smad3 is a key mediator in the canonical TGF-β signalling pathway. However, the potential role of Smad3 in the TGF-β1-dependent tumour microenvironment remains incompletely characterized. In this study, we demonstrated that Smad3 is essential for TGF-β1-mediated cancer progression, as both genetic deletion and pharmacological inhibition of Smad3 produced a significant inhibition of cancer growth, invasion and metastasis, resulting in a markedly improved survival rate in mouse models of LLC lung cancer and B16F10 melanoma. Increase in production and anticancer activities of NK cells but decrease in angiogenesis, Treg response and tumour-invasive activities were found in the Smad3-knockout or -inhibited tumour-bearing mice. It is suggested that TGF-β1-mediated pro-tumorigenic microenvironment can be shifted into a tumour-suppressive one by targeting the Smad3-dependent tumour microenvironment. We demonstrated that Smad3 is an important checkpoint for TGF-β-mediated cancer progression in the tumour microenvironment. Therefore, targeting Smad3-dependent tumour microenvironment may represent a novel and effective anticancer therapy.

Several mechanisms may be associated with the promoting effect of Smad3-dependent microenvironment on cancer progression, including angiogenesis, Treg response and tumour-invasive factors, as well as availability of NK cells. We found that genetic deletion or pharmacological inhibition of Smad3 significantly enhanced the production of NK cells (NK1.1^+^/NKp46^+^ cells) locally in the tumour microenvironment and systemically in the peripheral blood and splenic tissue. The effect of Smad3^−/−^ microenvironment on NK cell production was confirmed on a B16F10-rechallenged model, where increased NK1.1^+^ NKp46^+^ NK cells were observed in the tumour tissue, blood and spleen of rechallenged Smad3^−/−^ mice compared with the rechallenged Smad3^+/+^ control. These novel findings suggest that TGF-β may act via a Smad3-dependent mechanism to facilitate cancer progression by suppressing NK cell production. This was further confirmed by *in vitro* finding that deletion of Smad3 also enhanced the production of NKp46^+^ IFN-γ^+^ NK cells from bone marrow cells. The critical role of NK cell immunity in Smad3-dependent tumour microenvironment was further demonstrated by depleting NK cells from tumour-bearing Smad3^−/−^ mice with a neutralizing anti-NK1.1 antibody to restore the aggressive progression of melanoma. However, the use of anti-NK1.1 antibody may also deplete NKT cells; therefore, the specific role of Smad3 in NK cell production was further examined by adoptive transfer of Smad3^−/−^ or Smad3^+/+^ NK cells (NK1.1^+^) into the tumour-bearing NK cell deficiency mice (NOD/SCID). We demonstrated an enhanced anticancer effects of Smad3^−/−^ NK cells in cancer progression whereby mice infused with Smad3^−/−^ NK1.1^+^ cells displayed reduced tumour growth compared with Smad3^+/+^ NK1.1^+^ cell-recipient mice. It has been reported that TGF-β can inhibit NK cell-mediated anticancer immunity, including maturation, cytotoxicity, IFN-γ production and the expression levels of activating receptors NKG2D and NKp46 (refs 15, 16, 17, 30, 31, 32, 33, 34). TGF-β can also suppress CD16-mediated NK cell IFN-γ production and antibody-dependent cellular cytotoxicity in human NK cells via Smad3 (refs 17). Our findings reveal an unexplored role of Smad3 in the suppression of NK cell production and activities. Results from this study may also provide insights to explain the observation that NK cell development and activities are largely impaired in patients with cancer^34^,^35^,^36^.

Another novel finding from this study was that TGF-β1 can directly inhibit the NK cell immunity via a Smad3–E4BP4 axis and facilitate the cancer progression. E4BP4 (NFIL3) is one of the important master transcription factors for NK cell differentiation^22^,^23^,^24^. Here we found that TGF-β1 downregulated E4BP4 expression in the bone marrow- and spleen-derived NK cells in a Smad3-dependent manner, whereas genetic deletion or pharmacological inhibition of Smad3 successfully inhibited the TGF-β1-mediated E4BP4 suppression in NK cells. More importantly, we identified that E4BP4 is a direct Smad3 target gene. The conserved SBS located in the UTR of E4BP4 gene was validated by ChIP assay. Smad3 could physically bind to the E4BP4 gene and negatively regulate its transcription in response to TGF-β1, which was further confirmed by the real-time PCR and luciferase reporter assays. The Smad3-mediated suppression of E4BP4 was functionally important not only in NK cell development but also in the cancer-killing activity. Knockdown of E4BP4 decelerated the differentiation of both CD244^+^ NKp46^+^ (immature NK cells) and CD244^+^ NKp46^−^ (NK cell progenitors) and also reduced the expression of INF-γ of NK cells from either Smad3^−/−^ or Smad3^+/+^ bone marrow cells. Interestingly, comparing to the study with transfer of Smad3^−/−^ T-bet knockdown NK cells, adoptive transfer of Smad3^−/−^ E4BP4 knockdown NK cells significantly promoted tumour growth in NOD/SCID mice with lower levels of IFN-γ production. This suggested that E4BP4 may be a more important transcriptional factor than T-bet in NK cell development and functions. The ability of adoptive transfer of E4BP4 knockdown Smad3^−/−^ NK cells to reverse the suppressive effect of Smad3^−/−^ NK cells on tumour growth in B16F10 tumour-bearing NOD/SCID mice revealed the specific role of E4BP4 in NK cell-mediated antitumour activities. This finding is consistent with a previous study that deletion of T-bet did not alter the IFN-γ expression by NK cells in response to parasitic infection^37^. A direct E4BP4-binding site can be predicted on the promoter of IFN-γ and the transcription of IFN-γ can be reduced in Smad3^−/−^ NK cells by E4BP4 knockdown in a T-bet independent manner. Therefore, it is suggested that E4BP4 may be one of the possible candidates of T-bet-independent pathway for Smad3-mediated NK cell immunity. Our study demonstrated that Smad3-mediated inhibition of E4BP4 transcription may be a critical mechanism by which deletion of Smad3 promotes NK cell immunity and suppresses cancer progression. Nevertheless, further investigations should be done to confirm the T-bet independent pathway of TGF-β/Smad3-mediated suppression on NK cell immunity.

Interestingly, we found that a majority of NK cells in the Smad3^−/−^ tumour microenvironment originated from bone marrow, as significant induction of GFP^+^ NK1.1^+^ NK cells was observed in the tumour tissues of the chimeric GFP^+^ Smad3^−/−^mice. Bone marrow is a primary site for NK cell development, and it offers the cellular substrates and soluble factors required for NK cell maturation^38^. NK cells can migrate from the bone marrow to peripheral lymphoid organs, such as the spleen and lymph nodes^38^. NK cells exert promising anticancer activity, which may be circumvent by tumour-friendly microenvironment via TGF-β/Smad3 signalling as defined in the present work. In line with this notion, we found that cancer cells (B16F10 and LLC) proliferated at the same rate in both Smad3^+/+^ and Smad3^−/−^ mice during the first week after tumour implantation, but the progression was dramatically inhibited in the Smad3^−/−^ mice from week 2 onwards. This interesting phenomena implies that the host anticancer immunity, presumably NK cells derived from bone marrow, was exclusively suppressed in the Smad3^+/+^ mice after the tumour was fully established; but this suppressive effect can be abolished when Smad3 was deleted or inhibited in the tumour microenvironment. This may explain the dramatic increase in GFP^+^ NK1.1^+^ NK cells at the tumour stroma of the GFP^+^ Smad3^−/−^ chimeric mice. These findings also indicate that NK cell development can be strongly suppressed in the bone marrow compartment of Smad3^+/+^ microenvironment during cancer progression, but it can be restored if Smad3 is eliminated. Thus Smad3 may function as a determining factor for NK cell development in the bone marrow, possibly via the TGF-β1/Smad3–E4BP4 axis as discovered in this study.

Besides the enhanced NK cell immunity, we also observed that upregulation of angiogenesis markers (for example, VEGF and CD31^+^ vessels) and cancer invasive and metastatic proteins (for example, MMP-2, MMP-9, MMP-13 and CXCR4) were largely inhibited in the tumour microenvironment of Smad3^−/−^ mice and in Smad3^+/+^ mice treated with SIS3. These findings were consistent with the reported properties of TGF-β/Smad3 signalling in the induction of VEGF and MMP expression^39^,^40^. It is well documented that both angiogenesis and cancer invasive and metastatic activities play a critical role in cancer progression^41^,^42^,^43^. Therefore, inhibition of angiogenesis and tumour-invasive activities may account for the additional anticancer mechanism by disrupting Smad3 in the tumour microenvironments. Furthermore, inhibition of CD4^+^ Foxp3^+^ Treg cells could be another mechanism associated with enhanced NK cell immunity in the tumour-bearing Smad3^−/−^ mice. It is well accepted that Smad3 is an important transcriptional factor that binds to Foxp3 and facilitates Treg development^12^,^13^. It is also reported that Treg cells within the tumour microenvironment can suppress cytotoxicity of NK cells, which is reversed by Treg targeted treatment^44^,^45^. Thus it is likely that enhancement of NK cell-mediated anticancer immunity may be associated with inhibition of Treg cells in the Smad3^−/−^ tumour microenvironment. However, the precise mechanism needs to be further investigated.

Moreover, the present study also demonstrated that targeting Smad3-dependent microenvironment may represent a novel and effective anticancer therapy. By using a SIS3 that specifically inhibits phosphorylation, DNA binding and protein interaction of Smad3^29^, we demonstrated that systemic treatment with SIS3 (2.5, 5, 10 μg g^−1^, intraperitoneal (i.p.), daily) successfully blocked the Smad3 activation in the tumour microenvironment and significantly suppressed the tumour growth and invasion and associated with a 100% overall survival rate in the B16F10 and LLC tumour-bearing Smad3^+/+^ mice. In line with the observations in the Smad3^−/−^ mice, SIS3 treatment also markedly enhanced the development and anticancer activities of NK cells via a E4BP4-dependent mechanism. This was supported by the finding that SIS3 treatment was capable of increasing E4BP4 expression in Smad3^+/+^ bone marrow and NK cells and reversed the suppressive effect of Smad3 on NK cell development. In addition, the suppressive effect of SIS3 on cancer progression was also associated with inhibition of angiogenesis (CD31 and VEGF) and tumour-invasive and metastatic activities, including the expression of MMP-2, MMP-9, MMP-13 and CXCR4. It is also possible that direct cytotoxicity of SIS3 on cancer cells *in vitro* may account for an additional antitumour effect of SIS3 *in vivo.* The findings suggested SIS3 as a potential checkpoint inhibitor for cancer. Thus systemic Smad3-targeted therapy may represent a novel and effective therapeutic strategy for invasive cancer.

In conclusion, Smad3-dependent tumour microenvironment plays a crucial role in cancer growth, invasion and metastasis. TGF-β establishes its tumour-friendly microenvironment by suppressing NK cell production and cancer-killing activity via a Smad3–E4BP4 axis. Our findings suggest that modulating the TGF-β-dependent tumour microenvironments by targeting Smad3 may represent an effective anticancer therapy.

## Methods

### Establishment of LLC-luc and B16F10-luc tumour cell lines

The murine lung adenocarcinoma LLC (CRL-1642, ATCC) and melanoma B16F10 (CRL-6475, ATCC) cells were cultured in DMEM supplemented with 10% heat-inactivated FCS (GIBCO-BRL), 1 μM sodium pyruvate, 2 mM glutamine, 100 U ml^−1^ penicillin G and 100 μg ml^−1^ streptomycin. The luciferase overexpression stable cell lines of LLC-luc and B16F10-luc were established by transfection of pRRL-CMV lentiviral-luciferase vector into the cancer cells, followed by clone selection.

### Syngeneic mouse tumour models

Littermate Smad3^−/−^ (deletion of exon 8 and disruption of exon 7) or Smad3^+/+^ mice on C57BL/6J (H-2b) background (both sexes, aged 6–8 weeks, 20–25 g) were kindly provided by Dr Chuxia Deng^10^ and were used in this study. Two syngeneic mouse tumour models were induced in Smad3^−/−^ and Smad3^+/+^ mice by subcutaneously (s.c.) inoculating 2 × 10^6^ LLC-luc or B16F10-luc cells (both from C57BL/6J mice) into the right flank of the mouse. For tumour rechallenge model, first B16F10 tumour were induced in Smad3^−/−^ and Smad3^+/+^ mice by s.c. inoculating 1 × 10^6^ B16F10-luc cells into the left flank of the mice, and the established tumours were surgically removed on Day 15. Three weeks after, second B16F10 tumours (1 × 10^6^ cells per mice, s.c.) were induced on the right flank of the same mice, tumour volumes were monitored and samples were collected on Days 15 and 20 for further analysis according to the protocol of Zaharoff *et al*.^46^. For metastasis study, 3 × 10^5^ of LLC-luc or B16F10-luc cells were i.v. administered into Smad3^−/−^ and Smad3^+/+^ mice. The number and volume of surface nodules of B16F10 melanoma (dark black) in the lung of each mouse were counted, while LLC-luc tumours were observed by bioluminescence imaging with IVIS Spectrum system (Caliper, Xenogen). The photon intensity from specific regions was quantified by using the Living Imaging software 4.2 (PerkinElmer). The tumour size was monitored every week with a Vernier caliper and tumour volume was calculated by the formula: *V* (in mm^3^)=0.5(*ab*^2^), where *a* is the long diameter and *b* is the short diameter as previously described^47^. The raw data underlying the tumour volume measurements are shown in Supplementary Data 1. Mice were observed daily for the weight loss, distress, food intake and activities. Mice were killed before the end point if >20% weight loss occurred or the tumour reached the maximum permitted size (2,000 mm^3^), but experiments could be extended under veterinary supervision under special circumstances, for example, if tumours grew faster than expected. The specific experimental approach and the data obtained have been reviewed by the Animal Ethics Experimental Committee at the Chinese University of Hong Kong and confirmed to be in accordance with local regulations. The survival rate was recorded over the entire experimental period and converted into prolonged survival following the Kaplan–Meier method^48^. All experimental procedures were approved by the Animal Ethics Experimental Committee at the Chinese University of Hong Kong (No. 13/049/GFR).

### Tumour model in GFP-expressing bone marrow chimeric mice

To determine the origin and role of Smad3-dependent tumour microenvironments in cancer progression, GFP^+^ Smad3^−/−^ and GFP^+^ Smad3^+/+^ bone marrow-chimeric mice were generated by cross-breeding Smad3^+/−^ C57BL/6J mice with GFP transgenic C57BL/6J mice. A lethal irradiation (1500, cGy, Cs-irradiator) were applied on Smad3^+/+^ mice for bone marrow depletion and then 5 × 10^6^ of bone marrow cells from the GFP^+^ Smad3^−/−^ or GFP^+^ Smad3^+/+^ mice were i.v. administered into each mice 3 days after irradiation. The establishment of bone marrow chimera was confirmed at week 6 by flow cytometry analysis with 95% of peripheral blood cells being GFP^+^ cells. We then induced mouse tumour models in GFP^+^ Smad3^−/−^ and GFP^+^ Smad3^+/+^ mice by s.c. inoculating 2 × 10^6^ LLC-luc or B16F10-luc cells into the right flank of the mouse as described above. The raw data underlying the tumour volume measurements are shown in Supplementary Data 1. The animals were observed daily and the tumour size and survival rate were measured as described in the section on syngeneic tumour models. The experimental procedures were approved by the Animal Ethics Experimental Committee at the Chinese University of Hong Kong (No. 13/049/GFR).

### NK cell depletion and adoptive transfer models

For depletion of NK cells, Smad3^+/+^ and Smad3^−/−^ mice were pretreated with the anti-NK1.1 (PK136, BioXCell) or immunoglobulin G1 (IgG1) as negative control antibody (200 μg per mouse, i.p.) at Day −1. Then B16F10-luc cells (2 × 10^6^ cells) were s.c. inoculated into groups of five mice with or without NK1.1^+^ cell depletion on Day 0, followed by three additional antibody treatments (200 μg per mouse, i.p., weekly). The efficiency of NK cell depletion was confirmed with the absence of CD49b^+^ cells (staining with anti-CD49b antibody, DX5, eBioscience) by flow cytometric analysis of small blood samples collected from the tail vein of antibody-treated mice.

To determine the specific role of Smad3-dependent NK cell antitumour activities, a B16F10 tumour-bearing NOD/SCID mouse model^49^,^50^ was adoptively transferred with Smad3^+/+^ or Smad3^−/−^ NK cells (NK1.1^+^). Briefly, B16F10 cells (1 × 10^6^ cells) were inoculated s.c. into groups of three NOD/SCID mouse in which both NK and NK T cells are deficient. After tumour establishment on Day 5, Smad3^+/+^ NK1.1^+^ or Smad3^−/−^ NK1.1^+^ cells (5 × 10^6^ cells) were infused i.v. into the B16F10 tumour-bearing NOD/SCID mouse every 5 days with IL-2 supplement (i.p., 1,000 U per mouse) every 2 days for examining tumour growth and NK cell antitumour activities on Day 10.

In addition, we also compared the relative antitumour activities of E4BP4 versus T-bet-dependent NK cells by adoptive transfer of siE4BP4-Smad3^−/−^ NK1.1^+^ or siT-bet-Smad3^−/−^ NK1.1^+^ NK cells (5 × 10^6^ cells) into B16F10 tumour-bearing NOD/SCID mice as described above for examining tumour growth and NK cell antitumour activities. The raw data underlying the tumour volume measurements are shown in Supplementary Data 1. The animals were observed daily and the tumour size and survival rate were measured as described in the section on syngeneic tumour models. All experimental procedures were approved by Animal Ethics Experimental Committee at the Chinese University of Hong Kong (No. 13/049/GFR and 13/025/MIS).

### SIS3 treatment

The tumour-bearing Smad3^+/+^ mice were randomly divided into four groups (*n*=8 each group) on Day 7 after LLC-luc or B16F10-luc cancer cell (s.c.) inoculation. SIS3 (S0447, Sigma) at different dosages (2.5, 5 or 10 μg g^−1^, i.p., daily) were administered into the mice for 2 weeks, whereas control group were received solvent control (0.05% dimethyl sulfoxide) instead. Tumour size was monitored with a Vernier caliper every 2 day during the treatment. All mice were subjected to bioluminescence imaging and then killed after the survival rate was recorded on 15 days after SIS3 treatment. To determine the role of NK cells in SIS3-mediated tumour suppression, NK cell depletion was done on SIS3-treated mice and compared the result with SIS3-treated IgG control group on Day 15. The tumours were isolated, weighted and photo-recorded. Tumour tissues were collected for further analysis. The raw data underlying the tumour volume measurements are shown in Supplementary Data 1. The animals were observed daily and the tumour size and survival rate were measured as described in the section on syngeneic tumour models. All experimental procedures were approved by Animal Ethics Experimental Committee at the Chinese University of Hong Kong (No. 13/025/MIS).

### NK cell isolation

Splenic and bone marrow NK cells of the tumour-bearing mice were harvested by passing the cells through a 40 μm nylon mesh to produce a single-cell suspension. Erythrocyte contamination was eliminated by using RBC lysis buffer (Sigma) and the NK cells were isolated by using the NK Cell Isolation Kit II (Miltenyi Biotec), which provided 90–95% purity of NK cells as determined by flow cytometry with CD3^−^ CD49b^+^ cells.

### Flow cytometric analysis

For quantitative analysis of NK cell populations, tissue samples of the tumour-bearing mice were mechanically dissociated in chilled PBS (supplemented with 3% BSA) and gently mashed through a 40-μm nylon mesh to produce a single-cell suspension. NK cell populations were analysed with a flow cytometer by staining with Alexa 488-conjugated anti-mouse CD49b (DX5, eBioscience), PE-conjugated anti-mouse NK1.1 (PK136, eBioscience), Cy3-conjugated anti-mouse NKp46 (bs-2417R-cy3, Bioss) and PE-conjugated anti-mouse CD244 (12-2441-83, eBioscience) antibodies. For IFN-γ analysis, NK cells (1 × 10^6^) were cultured in the presence of 2 μM monensin (eBioscience) and then fixed and stained intracellular IFN-γ (eBioscience) for 5 h. The samples were subjected to acquire on FACSCalibur (Becton Dickinson) and analysed with the WinMDI 2.9 software and Cytobank platform (cytobank.org).

### NK cell cytotoxicity assay

The anticancer activity of NK cells was evaluated by using a Cella-Tox Bioluminescence Cytotoxicity Assay Kit (Cell Technology Inc.). In brief, isolated splenic NK cells from the tumour-bearing Smad3^−/−^ or Smad3^+/+^ mice were incubated with the NK-sensitive target cells (YAC-1), syngeneic LLC or B16F10 cells at ratios of 1.25:1, 2.5:1, 5:1, 10:1 and 20:1, respectively. In addition, splenic NK cells isolated from normal Smad3^+/+^ mice activated by 500 U ml^−1^ IL-2 for 3 days with or without SIS3 treatment or Day −8 Smad3^−/−^ or Smad3^+/+^ (with or without SIS3 treatment) bone marrow-derived NK cells were incubated with B16F10 cells. After 4-h incubation at 37 °C, cytotoxicity signals were detected by a luciferase bioluminescence method, and results were calculated according to the manufacturer's instructions.

### Western blotting analysis

Total proteins of tumour or normal (the skin of the same mouse) tissues were extracted by chilled RIPA lysis buffer (Pierce) and then subjected to the western blotting analysis with primary antibodies against CD31, VEGF, MMP-2, MMP-9, MMP-13, CXCR4, NKp46, p-Smad3, Smad3 (all from Santa Cruz Biotechnology) and E4BP4 (Cell Signaling) in 1:1,000, followed by incubation with the corresponding IRDyeTM800-conjugated secondary antibodies (1:10,000, Rockland Immunochemicals). β-Actin was used as an internal control. Expression levels of the proteins were detected by using LiCor/Odyssey infrared image system (LI-COR; Biosciences), and the band intensities were quantified with the Image J software (version 1.48, NIH, Bethesda). Images have been cropped for presentation; their full size images are presented in Supplementary Figs 15–20.

### Histology and immunohistochemistry

For immunohistochemistry, tumour tissues embedded in Optimal Cutting Temperature were cut into 6 μM sections and stained with the specific antibodies. The microvessels of tumour tissues were detected by anti-mouse CD31 antibody (BD Pharmingen) with Alexa Fluor 488-conjugated secondary antibody (Invitrogen), and the results were quantified as microvessel densities according to the method of Weidner *et al*.^51^. Treg cells in the tumour tissues were detected by immunofluorescence with fluorescein isothiocyanate (FITC)-conjugated anti-CD4 and Cy3-conjugated anti-Foxp3 antibodies (eBioscience). NK cells were identified by anti-mouse NK1.1 (PK136; R&D) and NKp46 (R&D) antibodies, while IFN-γ expressing NK cells were determined with Cy3- or APC-conjugated anti-NK1.1, FITC-conjugated anti-NKp46 and FITC- or APC-conjugated anti-IFN-γ antibodies (eBioscience). All antibodies were 1:100 in dilution. Cell nuclei were counterstained with DAPI (4,6-diamidino-2-phenylindole; Sigma). Positive cells and total cell counts from 10 high-power-field (× 40) tumour tissues were scored and expressed as a percentage of positive cells for each animal.

### Enzyme-linked immunosorbent assay

Levels of plasma and intratumoural granzyme B, IL-2 and IFN-γ were determined by using the commercial enzyme-linked immunosorbent assay kits (eBioscience). To collect the intratumoural tissue fluids, the chilled PBS was added into each tumour tissue samples in a ratio of 100 mg tissue per ml. The tissues were then homogenized and centrifuged at 14,000 r.p.m. at 4 °C for 10 min. The collected tumour tissue solutions and blood plasma were subjected for determination of NK cell activities, including granzyme B, IL-2 and IFN-γ, following the manufacturer's instruction.

### RNA extraction and real-time PCR

Total RNA was isolated from the cells using the RNeasy Isolation Kit (Qiagen, Valencia, CA) according to the manufacturer's instructions. Real-time PCR was performed using Bio-Rad iQ SYBR Green supermix with Opticon2 (Bio-Rad, Hercules, CA) as described previously^52^. The primers used in this study included: E4BP4 forward 5′-CAGTGCAGGTGACGAACATT-3′ and reverse 5′-TGTTCCACCACACCTGTTTTGA-3′; and GAPDH (glyceraldehyde 3-phosphate dehydrogenase) forward 5′-GCATGGCCTTCCGTGTTC-3′ and reverse 5′-GATGTCATCATACTTGGCAGGTTT-3′. The ratio for E4BP4 expression was normalized with GAPDH and expressed as the mean±s.e.m.

### siRNA knockdown

Smad3^−/−^ and Smad3^+/+^ bone marrow cells were harvested and transfected with (100 nM) nonsense control (siN05815122147, RIBOBIO) and siRNA against E4BP4 (5′-GAUGAGGGUGUAGUGGGCAAGUCUU-3′) on Days 0 and 4 as described previously^53^. The bone marrow cells were *ex vivo* differentiated into NK cells according to the protocol of Fathman *et al*.^54^. For adoptive transfer studies in NOD/SCID mice model, the *ex vivo* differentiated NK cells were transfected with nonsense control, siRNA against E4BP4 or T-bet (5′-GGGAGAACUUUGAGUCCAU-3′) on Day 5. The cells were collected on Day 6 (for Fig. 7) or Day 7 (for NK cell infusion, Supplementary Fig. 5). The maturation and cytotoxicity of NK cells were quantified by flow cytometry according to the CD244, NK1.1, NKp46 or IFN-γ expression profiles with directed dye-conjugated anti-mouse antibodies (eBioscience).

### MTT (methyl-thiazoldiphenyl tetrazolium) assay

The MTT assay was used to determine the antiproliferative activity of SIS3 on B16F10 cells *in vitro*. In brief, cancer cells (1 × 10^4^ per well) were seeded on a 96-well plate and serial concentrations of SIS3 (0, 2.5, 5, 10, 15, 20 μM) were added on the next day. After 24-h of SIS3 treatment, 30 μl of MTT (5 mg ml^−1^) was added to each well and incubated for 2 h at 37 °C. The MTT solution was then replaced by 100 μl of dimethyl sulfoxide in each well and measured with a microtitre-plate reader (Bio-Rad) at 540 nm, and all data were calculated as a percentage against the control.

### ChIP assay

ChIP was performed by using the SimpleChIP Enzymatic Chromatin IP Kit (magnetic beads; Cell Signaling) according to the manufacturer's instructions. In brief, bone marrow cells were isolated from Smad3^**+/+**^ mice pretreated with 1 ng ml^−1^ TGF-β1 for 2 h and then isolated by the Kit (Cell Signaling). Immunoprecipitation was performed with the antibody against Smad3 (1:100) and IgG was used as a control (Cell Signaling). Precipitated DNAs were identified by PCR using specific primers that detect the binding of Smad3 to 3′ UTR of mouse E4BP4 gene: forward 5′-CCTCTGACACATCGGAGAGC-3′ and reverse 5′-CGGAGAACAAGCTGATCGCCCT-3′.

### Luciferase reporter assay of E4BP4 3′ UTR

Construction of reporter and mutant plasmids and luciferase assay were performed by Landbiology (Guangzhou, China). For E4BP4 3′ UTR reporter, a pair of reporter plasmids was constructed with the SBS in E4BP4 3′ UTR or its mutant by inserting the following oligos (E4BP4XhoIF: 5′-ccgctcgagATGGCTGCTGACCGAGCTATGC-3′ and E4BP4NotIR:5′-ataagaatgcggccgcTGTGCTTGCCACCAATCCTTT-3′) or (mut-E4BP4_F:5′-GATGCCCTCACTCTGCCTGGCGTGTAAATTTGGGGCCCCCCACAGAGGCTGTACATACT-3′ and mut-E4BP4_R:5′-AGTATGTACAGCCTCTGTGGGGGGCCCCAAATTTACACGCCAGGCAGAGTGAGGGCATC-3′) into the psiCHECK-2 vector. In addition, a pair of overexpression plasmids was constructed with the complete cds of Smad3 (GI:2564492) or its mutant with the deletion of exon 8 by amplifying our Smad3 full-length pcDNA3.0 plasmid with the following primers: (mut-Smad3_F:5′-TGGGTTCCCCCAGCATCCGCTGTTCCGTGTAGAGACACTGGGAGTAAAGGGATCGGGT-3′ and mut-Smad3_R:5′-ACCCGATCCCTTTACTCCCAGTGTCTCTACACGGAACAGCGGATGCTGGGGGAACCCA-3′).

For dual luciferase reporter assay (Promega), the E4BP4 luciferase reporter plasmids and Smad3 plasmids were co-transfected into 293T cells. The luminescence in lysates of harvested cells was measured at 48 h according to the manufacturer's instructions. The luciferase activity (M1) and Renilla luciferase activity (M2) were measured by GloMax-Multi Detection System (Promega). The reporter activity was represented by the ratio of M1/M2, which was the normalized luciferase activity of the experimental plasmid (pGL4-basic; Promega). Results are shown as mean±s.d. fold induction of luciferase in at least three independent experiments.

### Statistical analysis

Statistical analysis of the differences in MTT assay, tumour volume, tumour weight, photon intensity, ratios of tumour nodules and positive cells, protein and mRNA expression levels, dual luciferase reporter assay and NK cell cytotoxicity were performed using analysis of variance, followed by Newman–Keuls Post Test from the Prism Program (Prism 5.0 GraphPad Software, San Diego, CA). Survival rates were generated based on the Kaplan–Meier method and statistical significance was determined by the log-rank test, *P* value <0.05 was considered statistically significant.

### Data availability

All data are available within the article, as figure source data or Supplementary Information Files, or from the authors upon reasonable request.

## Additional information

**How to cite this article:** Tang, P. M.-K. *et al*. Smad3 promotes cancer progression by inhibiting E4BP4-mediated NK cell development. *Nat. Commun.* **8,** 14677 doi: 10.1038/ncomms14677 (2017).

**Publisher's note:** Springer Nature remains neutral with regard to jurisdictional claims in published maps and institutional affiliations.

## Supplementary Material

Supplementary Data 1 Raw data underlying tumour volume measurements.

Supplementary Information Supplementary Figures.
