# Supplementary material for: Smad3 promotes cancer progression by inhibiting E4BP4-mediated NK cell development
Source: Nat Commun. 2017 Mar 6;8:14677. doi: 10.1038/ncomms14677 (PMC5343519; doi:10.1038/ncomms14677)
Supplement: Supplementary Information — Supplementary Figures. [file ncomms14677-s2.pdf]

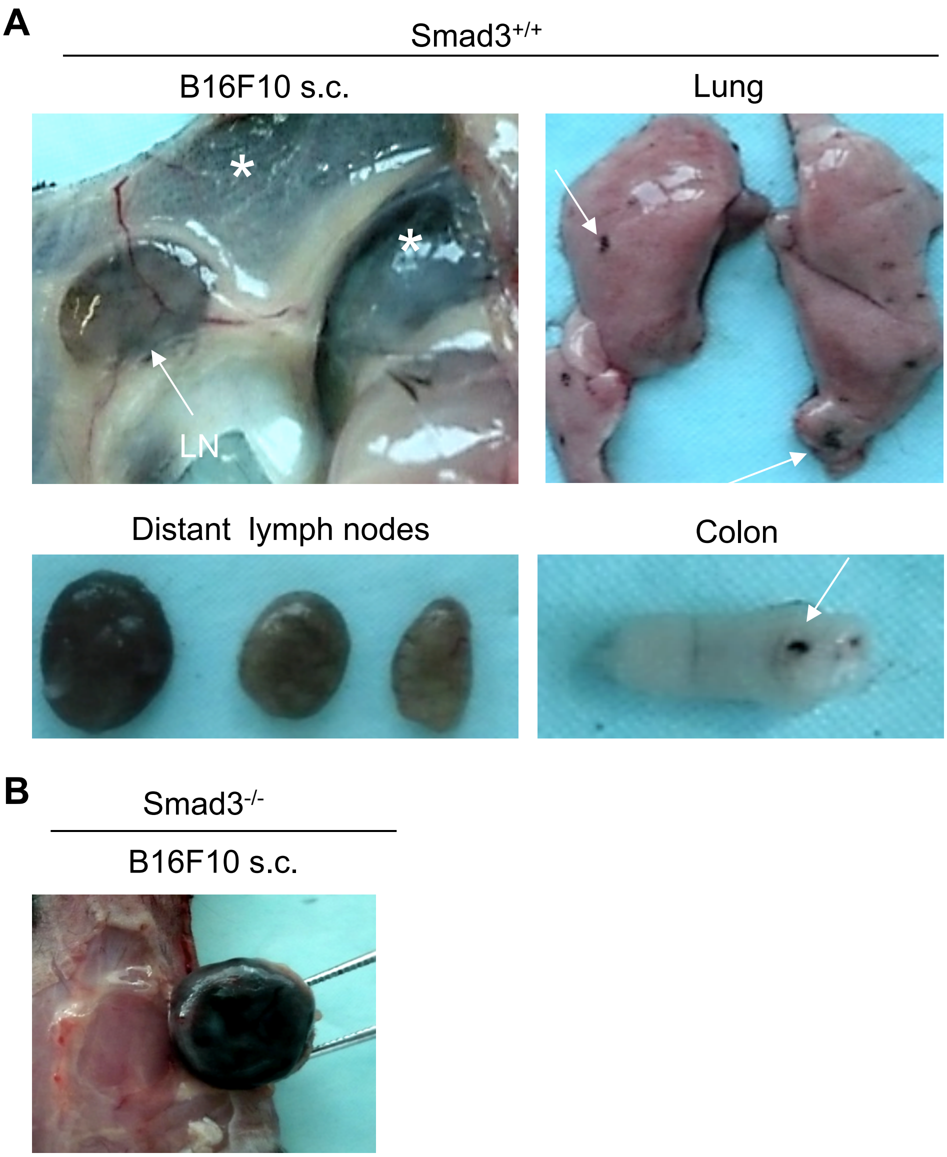

Supplementary Figure 1. Deletion of Smad3 prevents B16F10 melanoma invasion and metastasis in a mouse s.c. tumor model. Highly invasive growth patterns (*) and metastasis to lymph nodes (LN), lung (arrows), and colon (arrow) developed in B16F10 tumor-bearing Smad3$^{+/+}$ mice (A) are prevented in Smad3$^{-/-}$ mice (B). Representative macroscopic images are shown for groups of 8 mice.

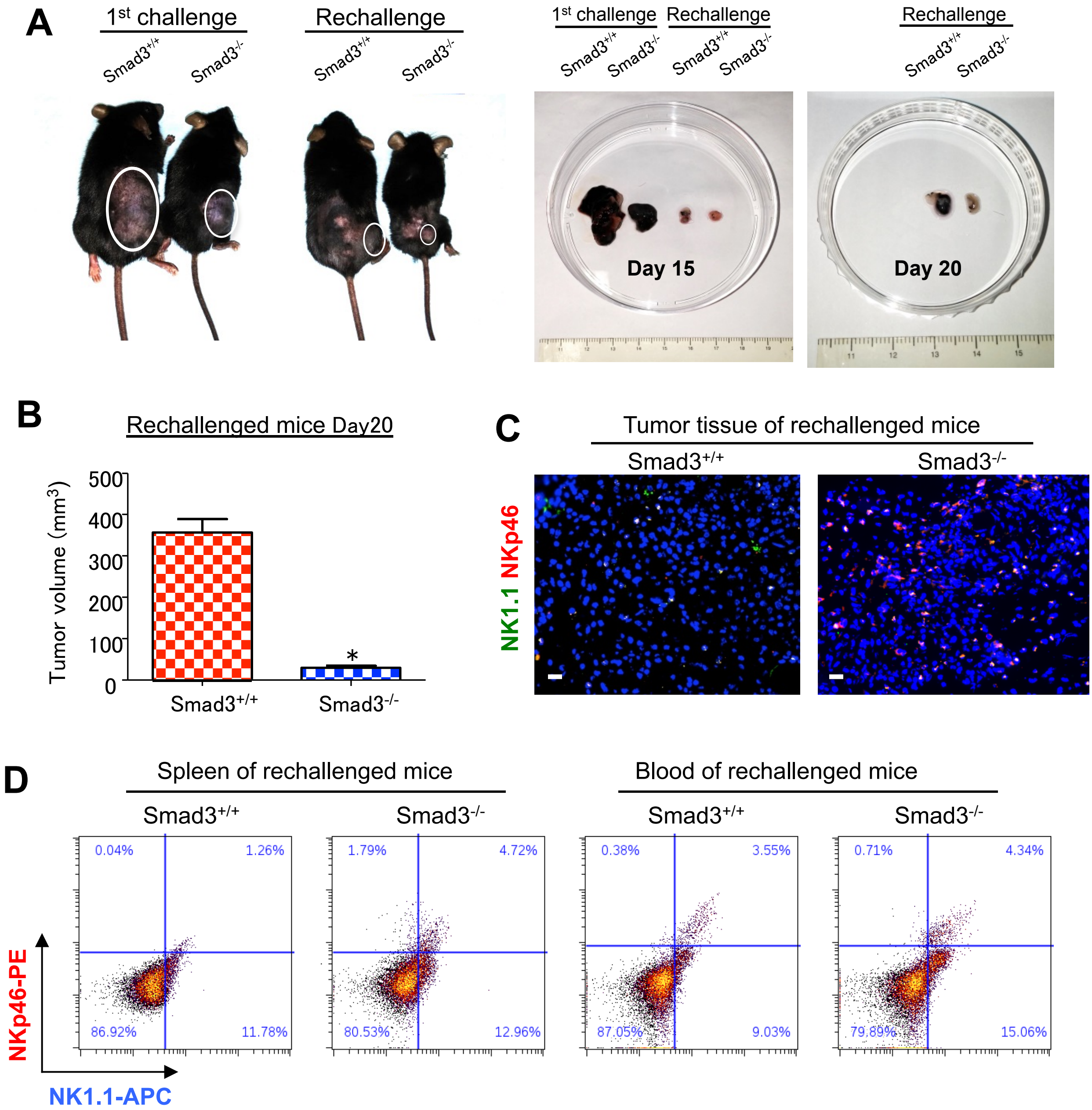

Supplementary Figure 2. Smad3$^{-/-}$ microenvironment reduces cancer progression in the tumor rechallenged mice. (**A** and **B**) The growth of B16F10 tumor was significantly suppressed in the Smad3$^{-/-}$ mice, qualified by imaging on Days 15$^{\#}$ and 20 (**A**) and quantified by the tumor volume on day 20 (**B**). (**C**) Tumor-infiltrating NK cells are largely increased in the rechallenged Smad3$^{-/-}$ mice compared to the Smad3$^{+/+}$ mice, which are showed by NKp46 and NK1.1 co-immunofluorescence imaging. (**D**) Mature NK cells (NKp46^{+}NK1.1^{+}) were increased in spleen and blood of the rechallenged Smad3$^{-/-}$ mice compared to the rechallenged Smad3$^{+/+}$ mice, which are quantified by 2-colour flow analysis of Day 15 samples. Data represent mean ± SEM for groups of 3 mice. *$p<0.05$, compared to B16F10 tumor-rechallenged Smad3$^{+/+}$ group analyzed by ANOVA. Scale bar, 100 μm. $^{\#}$ 1st challenged mice were scarified on Day 15 due to the restriction of maximum tumor volume (2000mm^{3}) under Animal Ethics Experimental Committee.

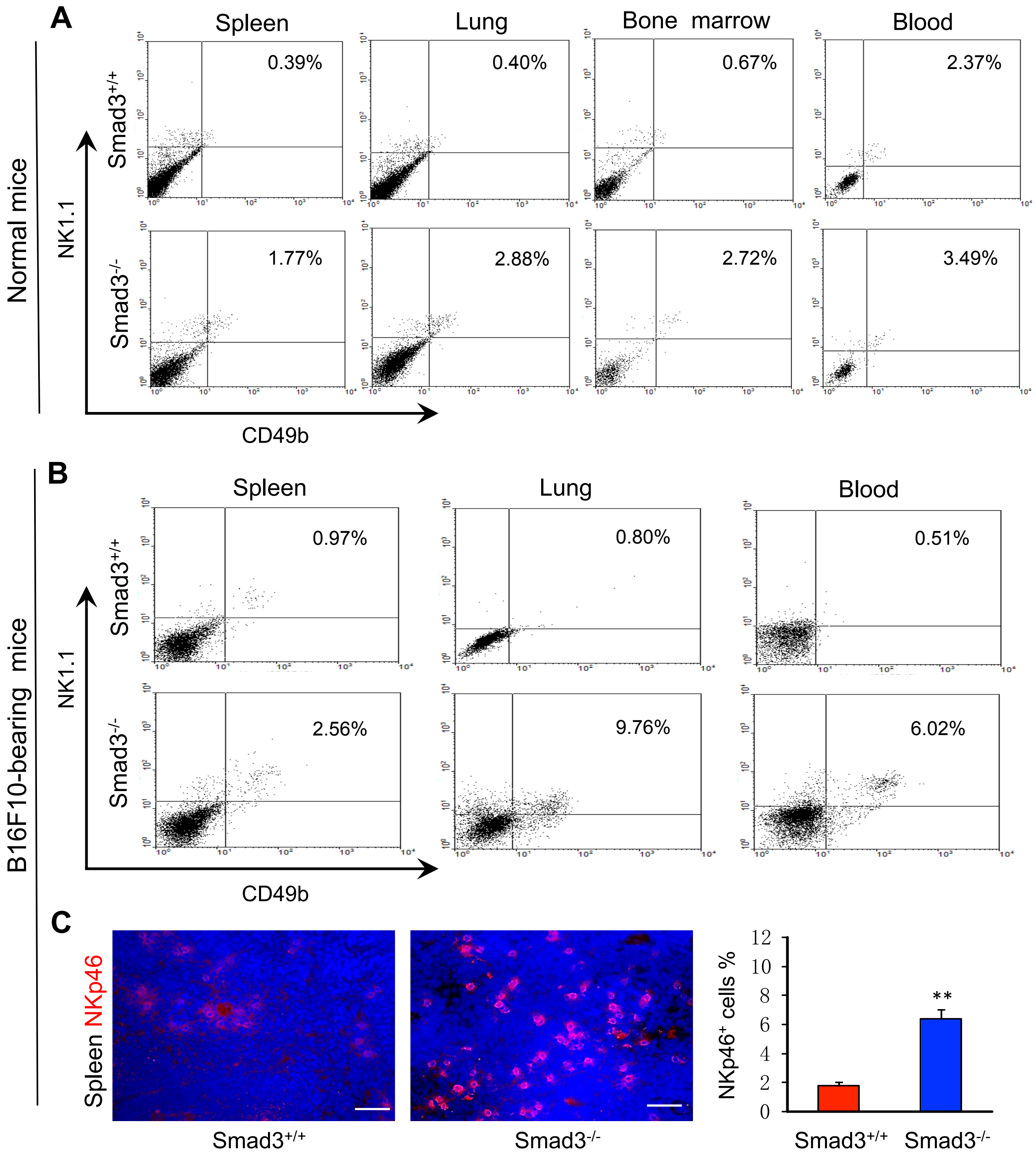

Supplementary Figure 3. Smad3-dependent microenvironment promotes cancer by suppressing the NK cell differentiation and maturation in normal and tumor-bearing mice. (**A** and **B**) Two-color flow cytometry shows that normal mice null for Smad3 develop higher levels of the NK1.1^{+} CD49b^{+} cell population in the spleen, lung, bone marrow, and peripheral blood (A), which is further increased in tumor-bearing Smad3$^{-/-}$ mice (up to a 10-fold increase in lung and peripheral blood), but not in Smad3$^{+/+}$ mice. (C) Increased splenic NKp46^{+} cells are detected in B16F10 tumor-bearing Smad3$^{-/-}$mice but not in Smad3$^{+/+}$ mice by immunofluorescence imaging. Data represent for groups of 8 mice. Scale bar, 100 μm.

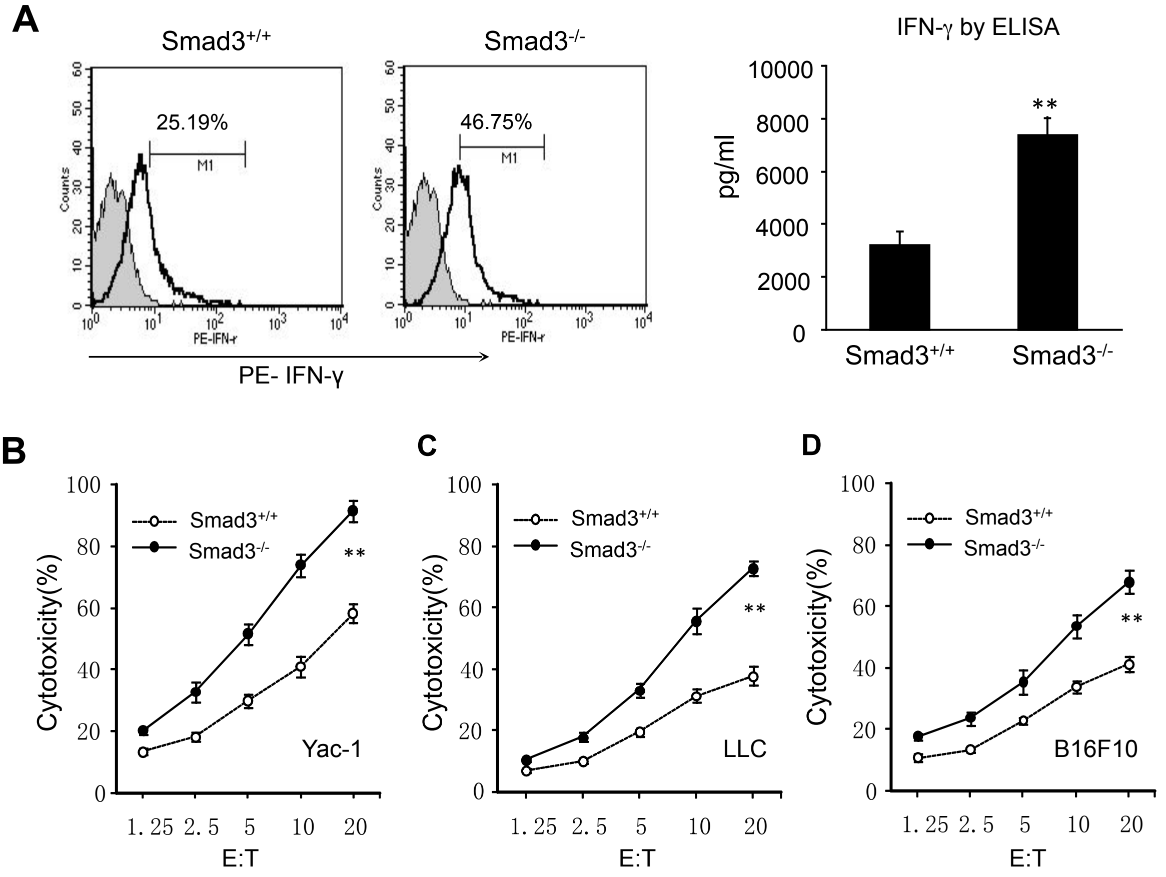

Supplementary Figure 4: Deletion of Smad3 enhances NK cell anti-cancer cytotoxicity *ex-vivo*. (**A**) Flow cytometry and ELISA show that IFN-γ-producing ability by splenic NKp46^{+} from tumor-bearing Smad3$^{-/-}$ mice is largely enhanced. (**B-D**) The cytotoxicity assay detects that deletion of Smad3 largely enhances the cell-killing activity by splenic NK cells against the NK-sensitive target cells YAC-1 (**B**), syngeneic LLC (**C**), and B16F10 (**D**) cancer cells. Data represent for mean ± SEM from 4 independent experiments. **p<0.01 compared between groups analyzed by ANOVA.

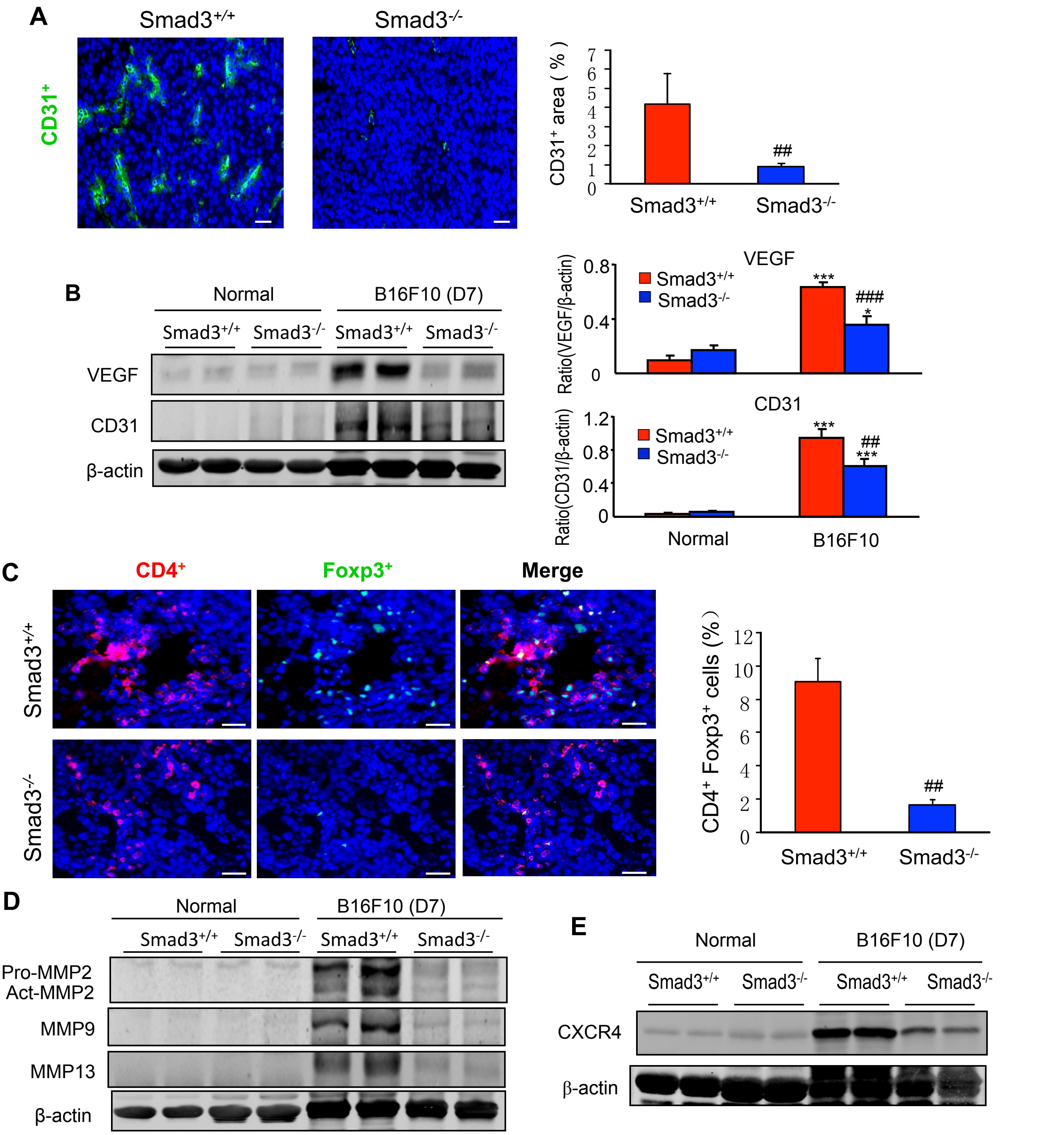

Supplementary Figure 5. Smad3 facilitates cancer progression by enhancing angiogenesis and Treg immune response in mice. (A) Immunofluorescence and (B) Western blot analysis detect a marked intratumoral angiogenesis identified by numerous CD31+ vessels and higher levels of VEGF and CD31 protein expression in B16F10 melanoma-bearing Smad3$^{+/+}$ mice, which is blunted in B16F10 melanoma-bearing Smad3$^{-/-}$ mice on day 7. (C) Two-color immunofluorescence shows that there are numerous CD4+ Foxp3+ cells infiltrating the tumor tissues of B16F10 melanoma-bearing Smad3$^{+/+}$ mice, which is absent in B16F10-bearing Smad3$^{-/-}$ mice. (D) Western blot analysis detect that there are high levels of intratumoral pro-and active MMP-2, MMP-9, and MMP-13 expression in B16F10 melanoma-bearing Smad3$^{+/+}$ mice, which is abolished in Smad3$^{-/-}$ mice. (E) Western blot analysis shows that mice null for Smad3 are prevented from a marked upregulation of intratumoral CXCR4 in the B16F10 melanoma tissues. Data represent mean ± SEM for groups of 8 mice. *p<0.05, **p<0.01 compared to normal; ##p<0.01, ###p<0.001 compared to tumor-bearing Smad3$^{+/+}$ mice analyzed by ANOVA. Scale bar, 50 and 100 μm.

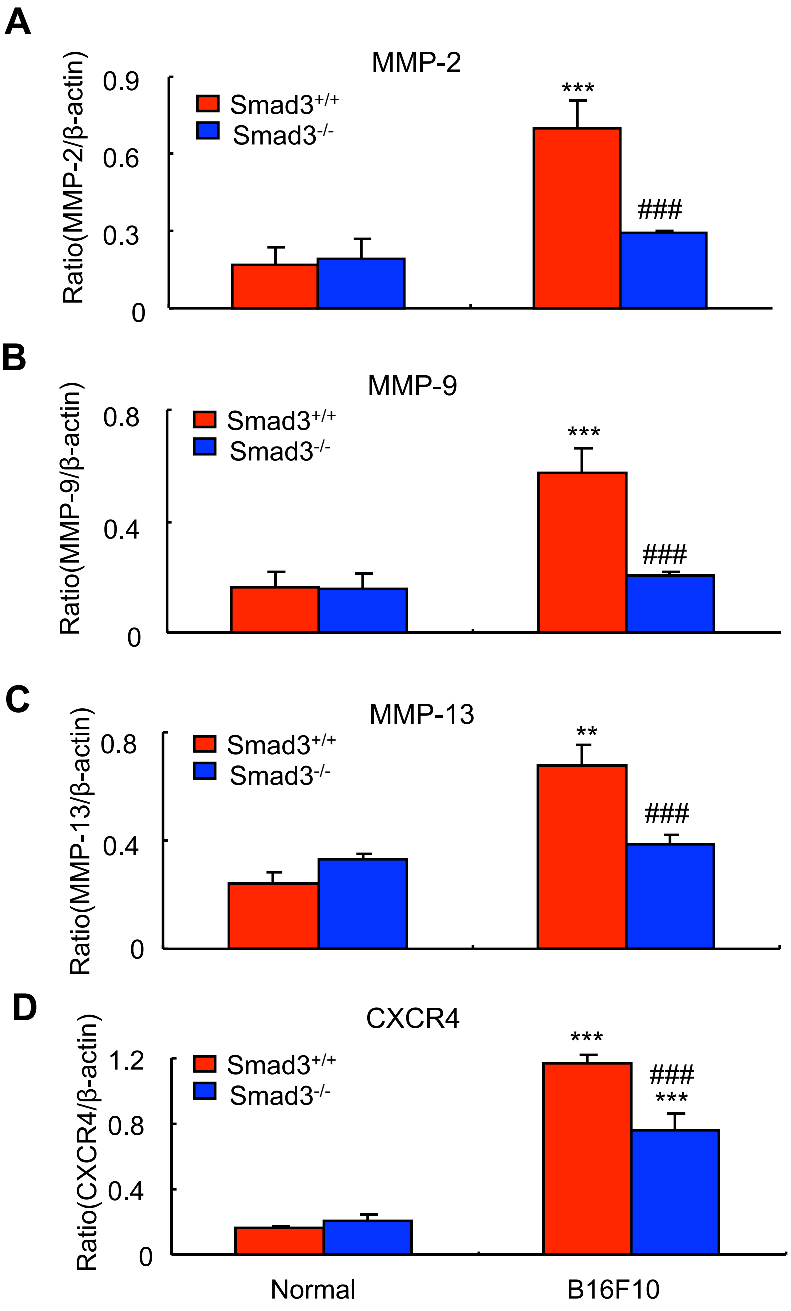

Supplementary Figure 6. Deletion of Smad3 suppresses MMPs-mediated matrix degradation and CXCR4 expression in B16F10 tumor bearing mice. (A) Quantification results of the Western blot analysis shows that higher levels of intratumoral pro- and active MMP-2, MMP-9, and MMP-13 expression in B16F10 melanoma-bearing $Smad3^{+/+}$ mice are protected in $Smad3^{-/-}$ mice. (B) Quantification results of the Western blot analysis shows that a marked upregulation of intratumoral CXCR4 in B16F10 melanoma-bearing $Smad3^{+/+}$ mice is prevented in $Smad3^{-/-}$ mice. Data represent mean ± SEM for groups of 8 mice. **p<0.01, ***p<0.001 compared to normal; ###p<0.001 compared to tumor-bearing $Smad3^{+/+}$ mice analyzed by ANOVA.

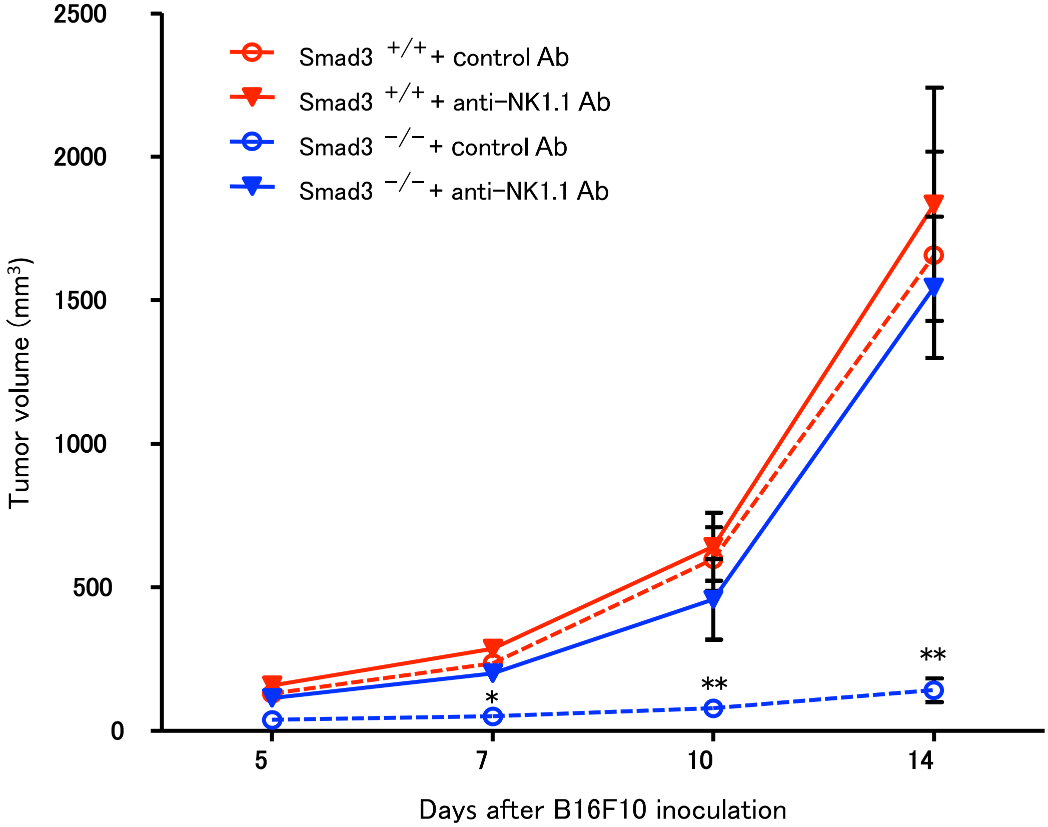

Supplementary Figure 7. NK cell depletion restores cancer progression in B16F10 tumor-bearing Smad3$^{-/-}$ mice but not in Smad3$^{+/+}$ mice. Tumor growth rate is largely inhibited in the Smad3$^{-/-}$ mice (blue), which is restored to the level of Smad3$^{+/+}$ mice (red) by depleting NK cells with the anti-NK1.1 antibody (Ab). In contrast, treatment with the anti-NK1.1 Ab produces no significant effect on the tumor growth rate on Smad3$^{+/+}$ mice. Data represent mean ± SEM for groups of 8 mice. *$p<0.05$, **$p<0.01$ compared to B16F10 tumor-bearing Smad3$^{+/+}$ mice analyzed by ANOVA.

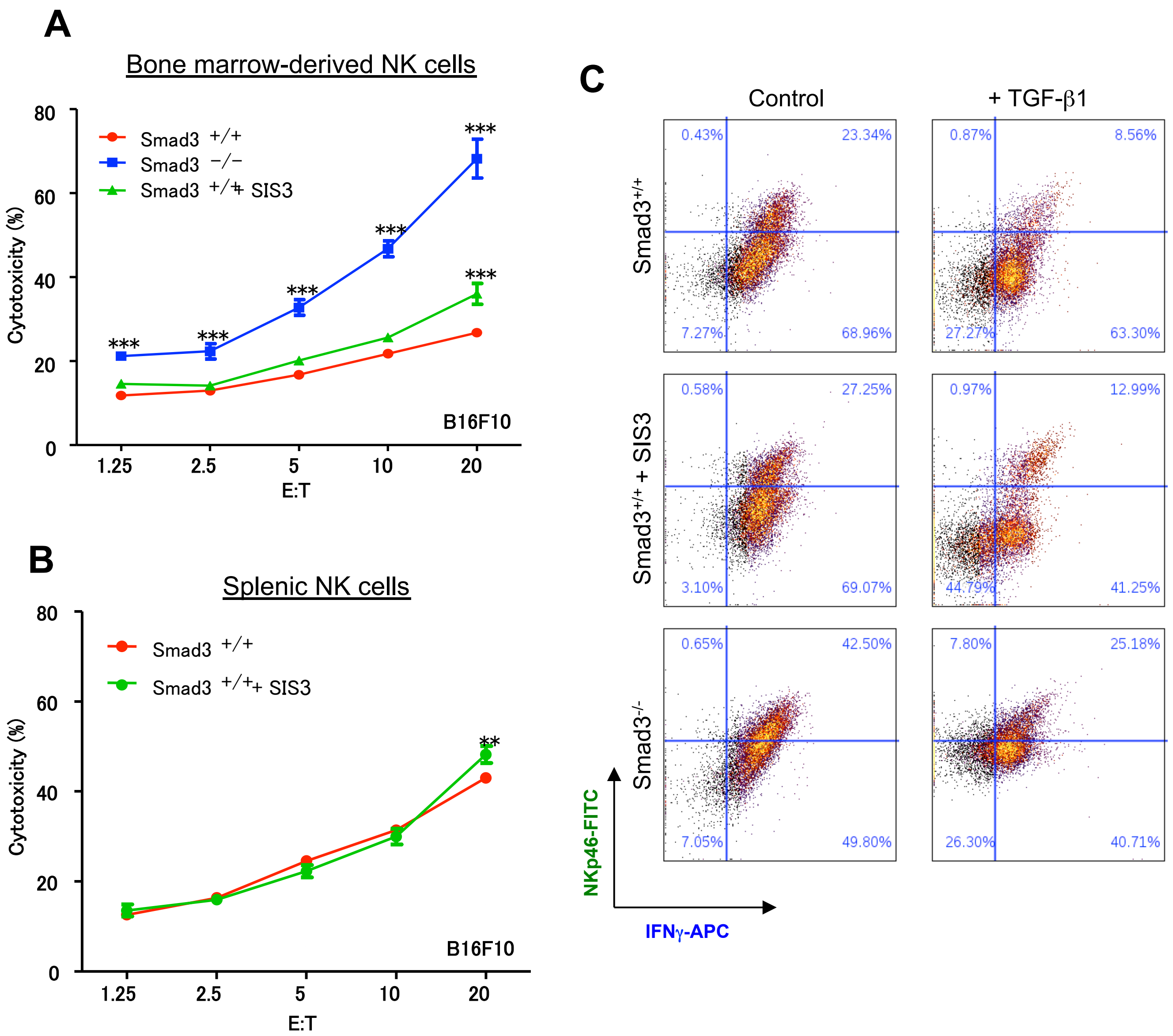

Supplementary Figure 8. Deletion and inhibition of Smad3 enhance the cancer killing activity of NK cells. (**A**) The cytotoxicity assay detects that deletion (Smad3$^{-/-}$) or inhibition (Smad3$^{+/+}$+SIS3, 1μM) of Smad3 largely enhances the cancer killing activity of bone marrow derived NK cells. (**B**) Smad3$^{+/+}$ splenic NK cells treated with SIS3 (1μM for 72h) promote tumor-killing activity of B16F10 melanoma cells. Data represent for mean ± SEM from 3 independent experiments. **p<0.01, ***p<0.001 compared to Smad3+/+ NK cells with SIS3 treatment analyzed by ANOVA. (**C**) Flow cytometry shows that deletion (Smad3$^{-/-}$) or inhibition (SIS3) of Smad3 largely increases the production of IFNγ^{+} NKp46^{+} bone marrow derived NK cells with or without TGF-β1 (0.5ng/ml), compared to control group (Smad3$^{+/+}$) on Day 8 NK differentiation *ex vivo*. Results shown are representative data of 3 independent experiments.

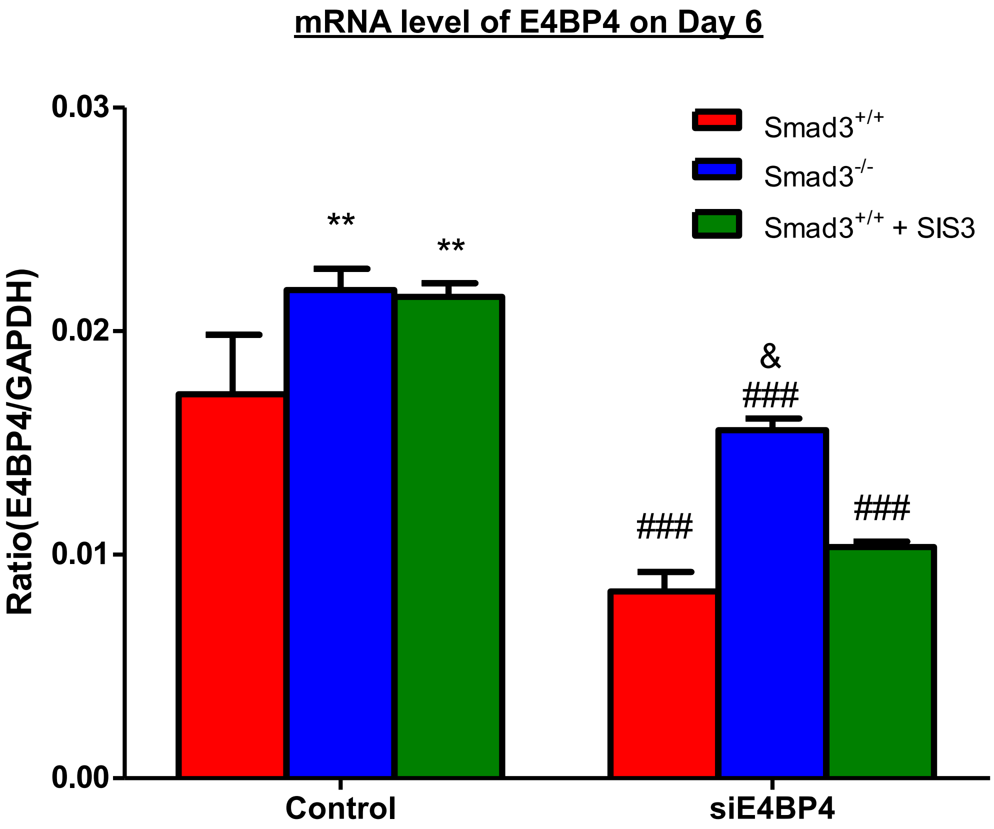

Supplementary Figure 9. Effect of siE4BP4 on the E4BP4 mRNA expression in bone marrow cells on Day 6. Bone marrow cells were transfected with nonsense (control) or siRNA against mouse E4BP4 (si-E4BP4), or treated with SIS3 (SIS3) on day 0 and day 4 and cells were cultured with the NK cell differentiation medium for 6 days. Expression of E4BP4 mRNA was measured by real-time PCR. Data represent mean ± SEM for 3 independent experiments. $^{**}p<0.05$ compared to Smad3$^{+/+}$ cells; $^{\#\#\#}p< 0.001$ compared to the individual control group before siE4BP4 treatment; $^{\&}p<0.05$ compared to Smad3$^{+/+}$ cells analyzed by ANOVA.

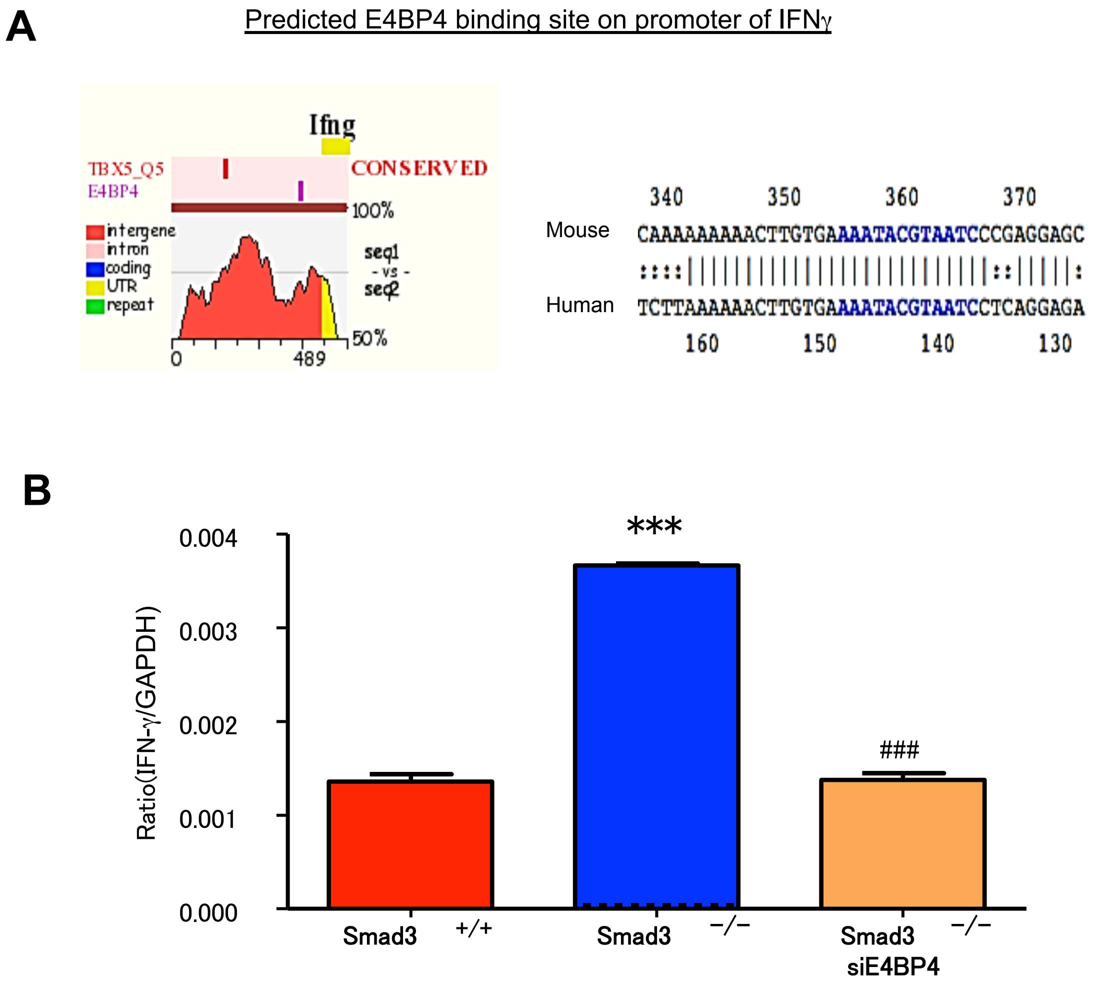

Supplementary Figure 10. E4BP4 knockdown reduces the enhancement of IFNγ transcription on Smad3$^{-/-}$ NK cells. (**A**) An E4BP4 binding site is predicted at the promoter of the evolutionarily conserved region of IFNγ (Ifng) nearby T-bet (TBX5_Q5) binding site in human and mouse genomes (left panel), the sequences of E4BP4 binding site is indicated as blue (right panel). (**B**) The increased mRNA levels of IFNγ in the *ex-vivo* differentiated Smad3$^{-/-}$ NK cells on Day 7 are largely suppressed by E4BP4 knockdown (siE4BP4). Results shown are representative data of 3 independent experiments, ***p<0.001 compared to the Smad3$^{+/+}$ cells and $^{###}$p<0.001 compared to Smad3$^{-/-}$ cells analyzed by ANOVA.

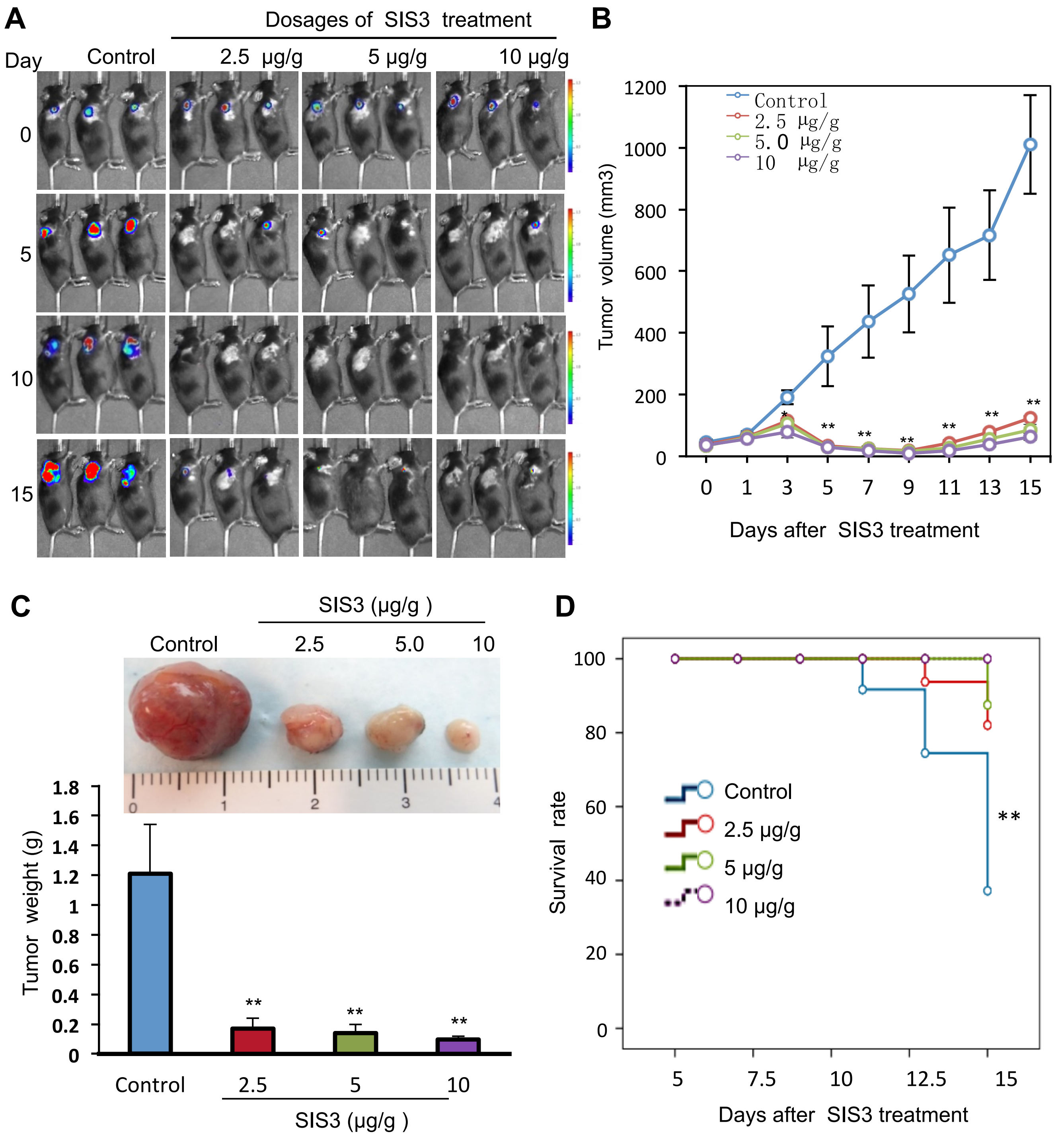

Supplementary Figure 11. SIS3 treatment inhibits the progression of invasive lung cancer LLC in Smad3$^{+/+}$ mice. LLC-luc tumor-bearing Smad3$^{+/+}$ mice treated with SIS3 are protected from the cancer growth and death in a dose-dependent manner as demonstrated by: the bioluminescent imaging (A), the tumor volumes (B), the tumor weights on day 15 after treatment (C), and the survival rates (D). Data represent mean ± SEM for groups of 8 mice. p<0.05, **p<0.01 compared to control-treated LLC-luc tumor-bearing Smad3$^{+/+}$ mice analyzed by ANOVA.

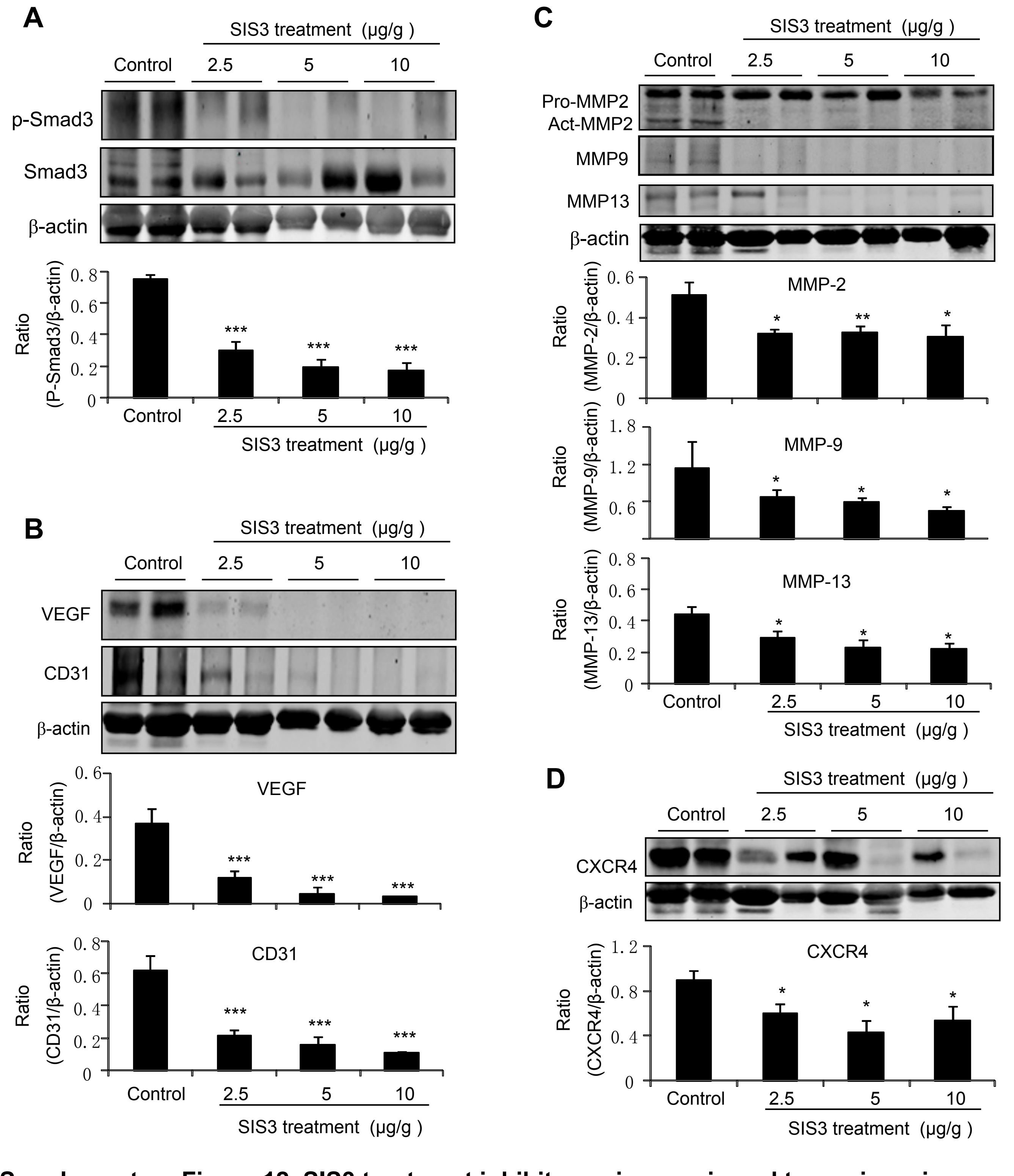

Supplementary Figure 12. SIS3 treatment inhibits angiogenesis and tumor invasive factors in LLC tumor bearing Smad3$^{+/+}$ mice. Western blot analysis shows that treatment with SIS3 dose-dependently inhibits intratumoral phosphorylation of Smad3 (**A**), angiogenesis including expression of VEGF and CD31 proteins (**B**), pro-and active MMP-2, MMP-9, and MMP-13 expression (**C**), and CXCR4 expression (**D**). Data represent mean ± SEM for groups of 8 mice. *p<0.05, **p<0.01, ***p<0.001 compared to the control-treated group analyzed by ANOVA.

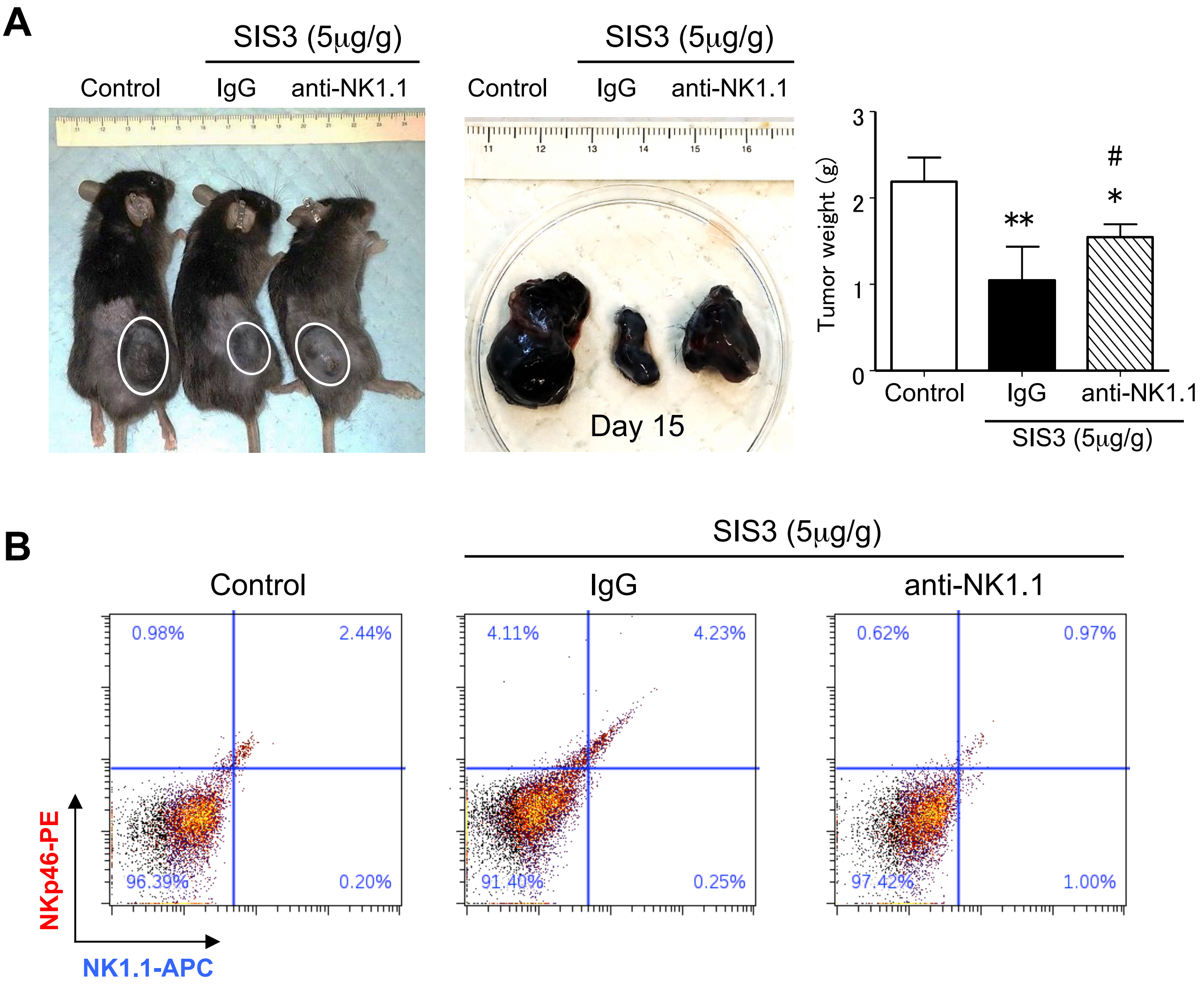

Supplementary Figure 13. Depletion of NK cells increases cancer progression in SIS3-treated Smad3$^{+/+}$ mice. (**A**) The growth of B16F10 tumor was significantly suppressed in the SIS3-treated IgG group (5μg/g/day, i.p.) compared to the untreated control group, which was partially reversed in the SIS3-treated anti-NK1.1 group (200μg/mouse, weekly i.v.) as qualified by imaging and tumor weight on day 15. (**B**) Mature NK cells (NKp46^{+}NK1.1^{+}) were increased in the blood of SIS3-treated IgG group compared to the untreated control group, but was largely reduced in the SIS3-treated anti-NK1.1 group as demonstrated by two-colour flow analysis of Day 15 samples. Data represent mean ± SEM for groups of 3 mice. *p<0.05, **p<0.01, compared to control group; $^{\#\#}$ p< 0.001, compared to the SIS3-treated IgG group analyzed by ANOVA.

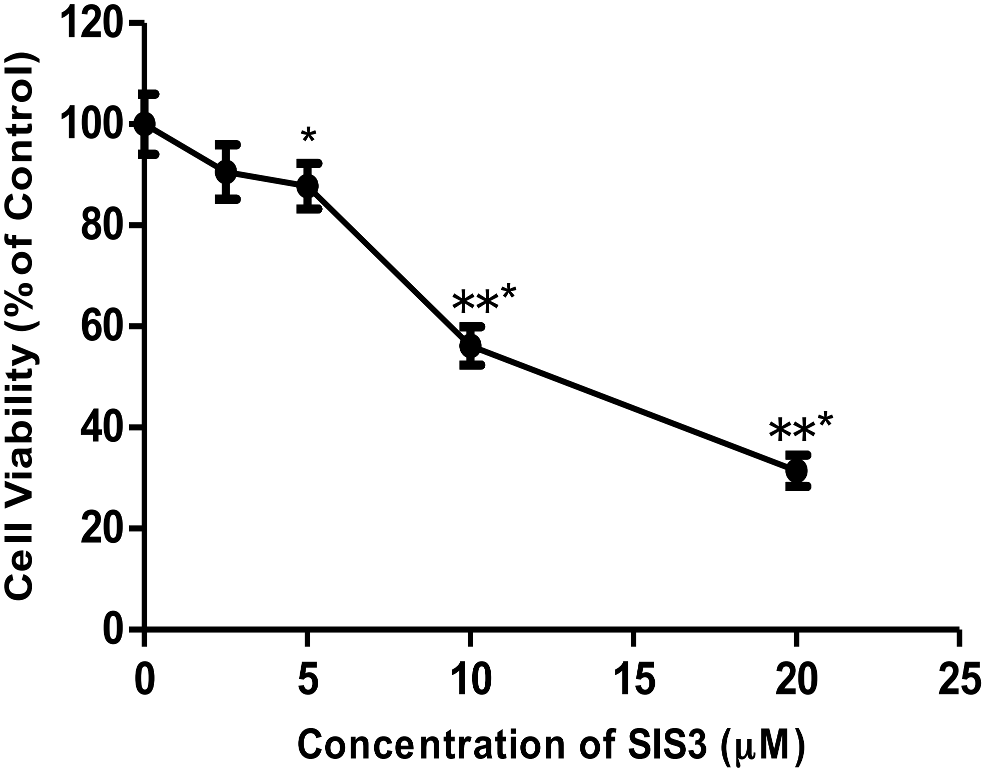

Supplementary Figure 14. Effect of SIS3 on proliferation of B16F10 cells. MTT assay shows that addition of SIS3 is able to inhibit B16F10 cell proliferation in a dose-dependent manner. Data represent mean ± SEM for 3 independent experiments. *p<0.05, ***p<0.001 compared to medium without SIS3 (0) analyzed by ANOVA.

Figure 2c

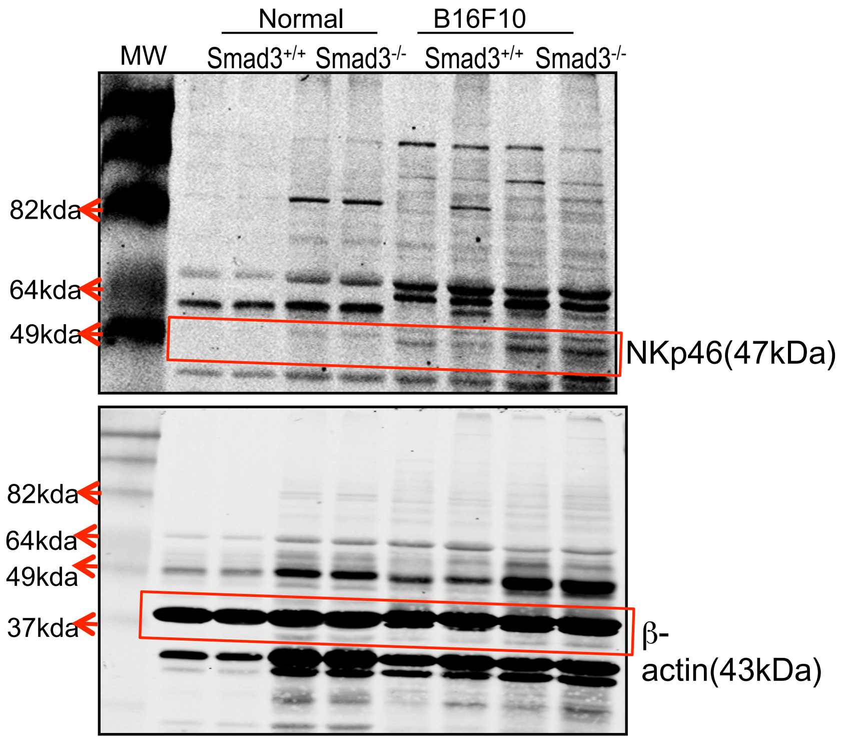

Figure 4a

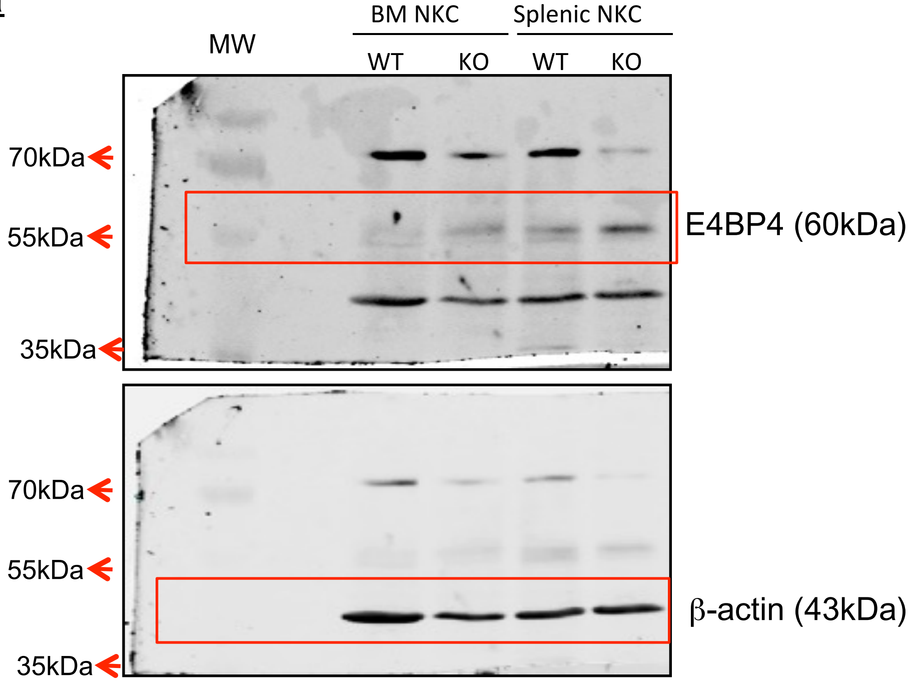

Supplementary Figure 15. Full-size scans of Western blots shown in Figures 2 and 4.

Figure 5d

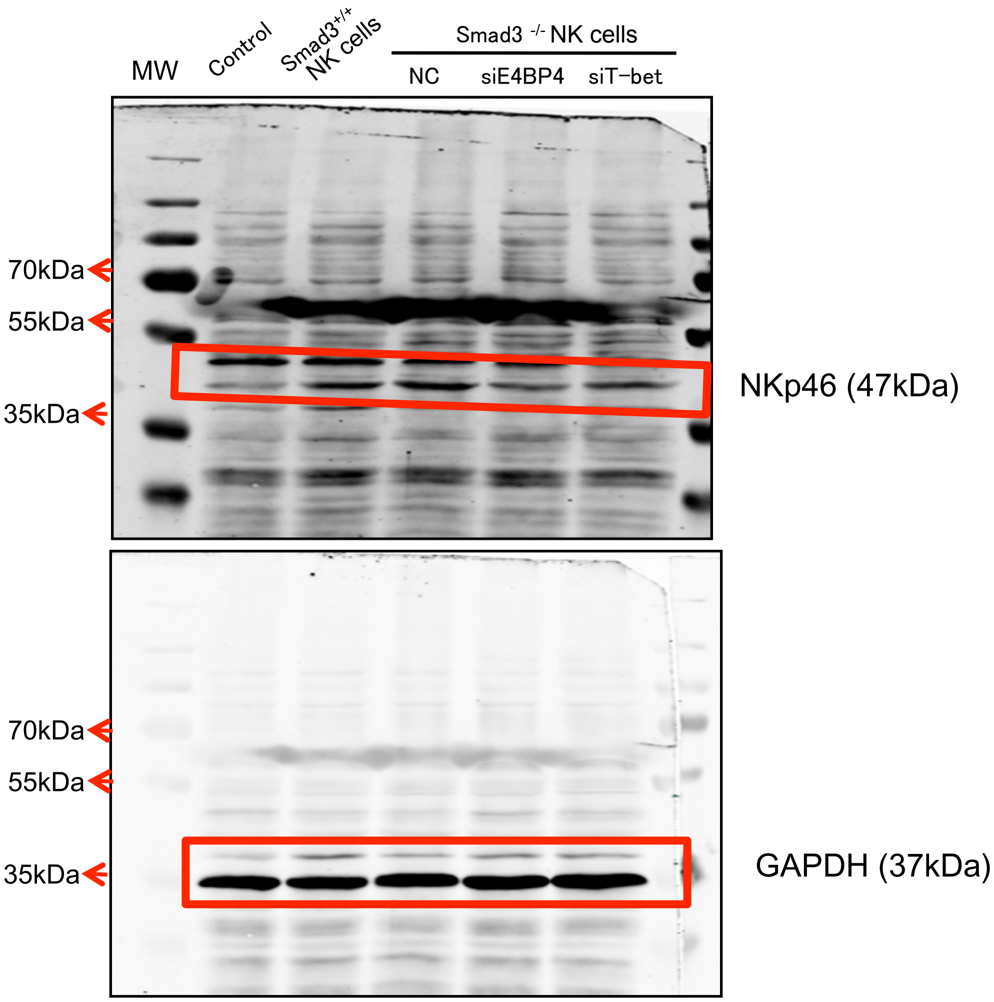

Supplementary Figure 16. Full-size scans of Western blots shown in Figure 5.

Figure 7a

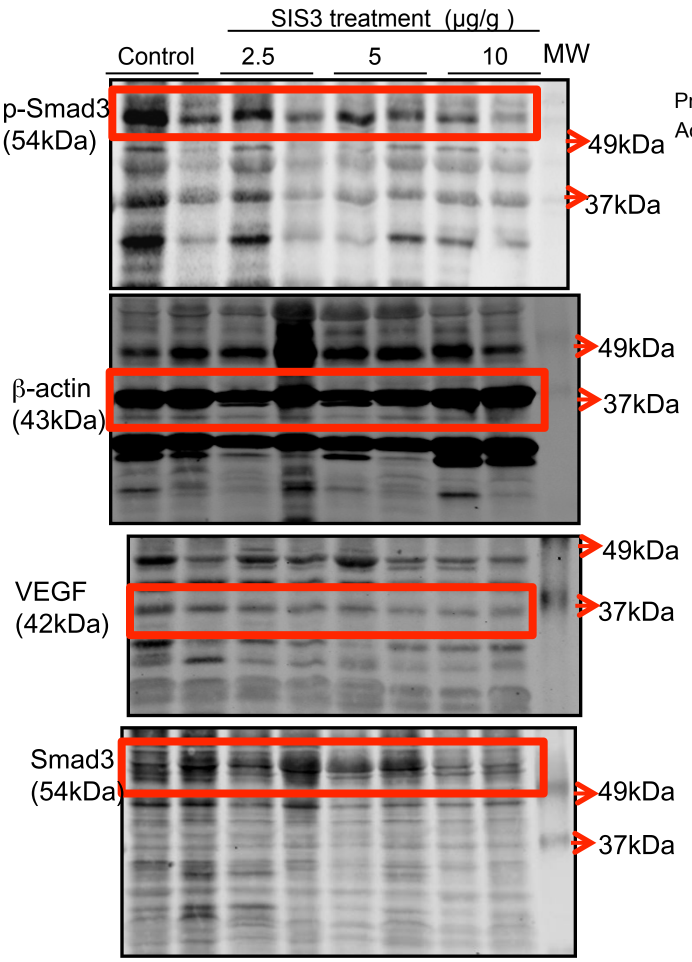

Figure 7b

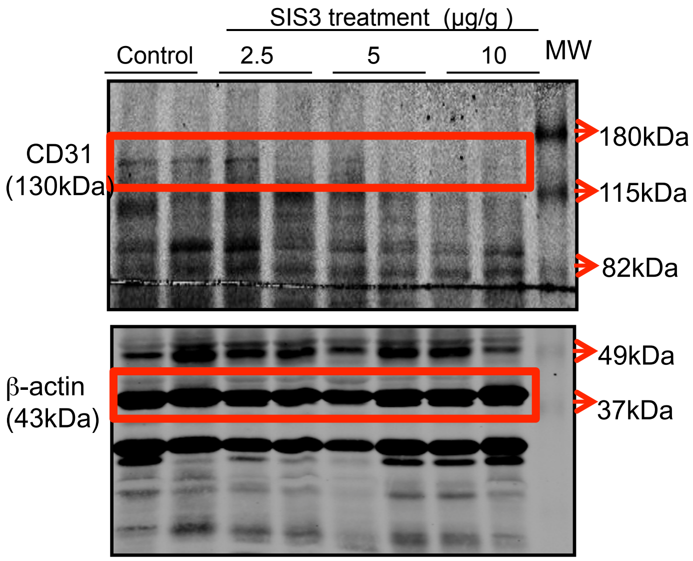

Figure 7c

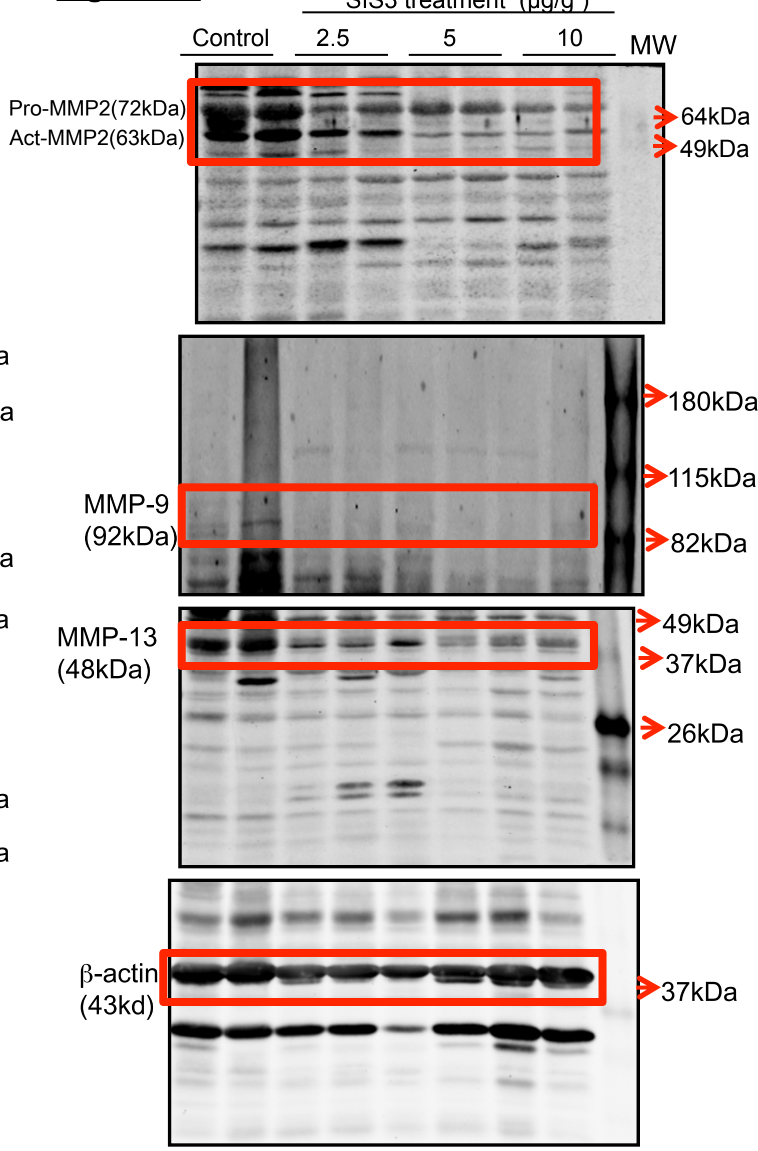

Figure 7d

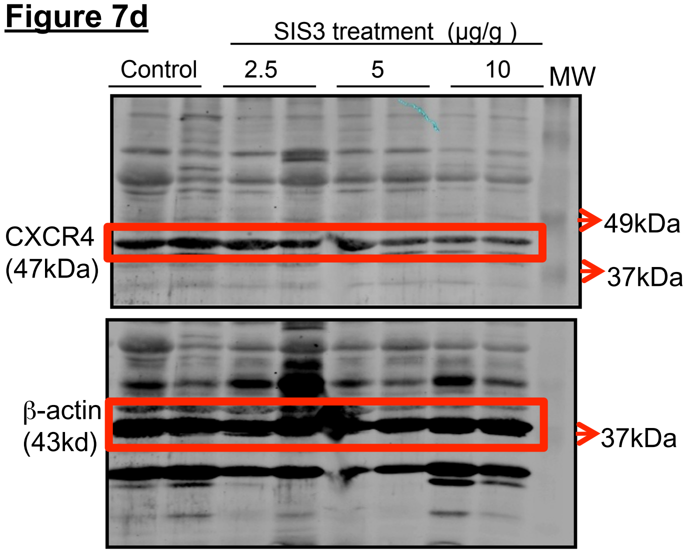

Supplementary Figure 17. Full-size scans of Western blots shown in Figure 7.

Supplementary Figure 5B

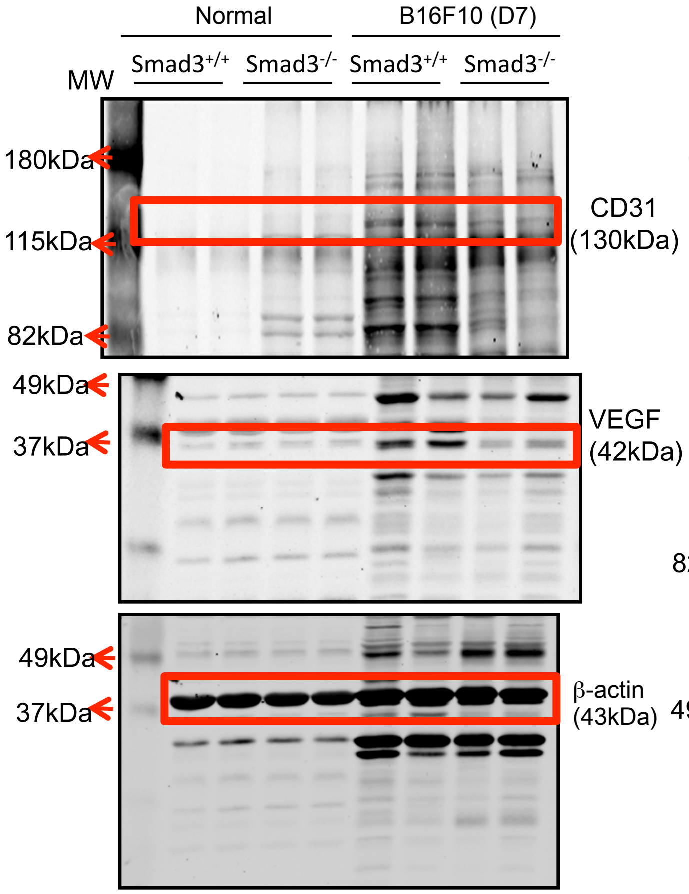

Supplementary Figure 5D

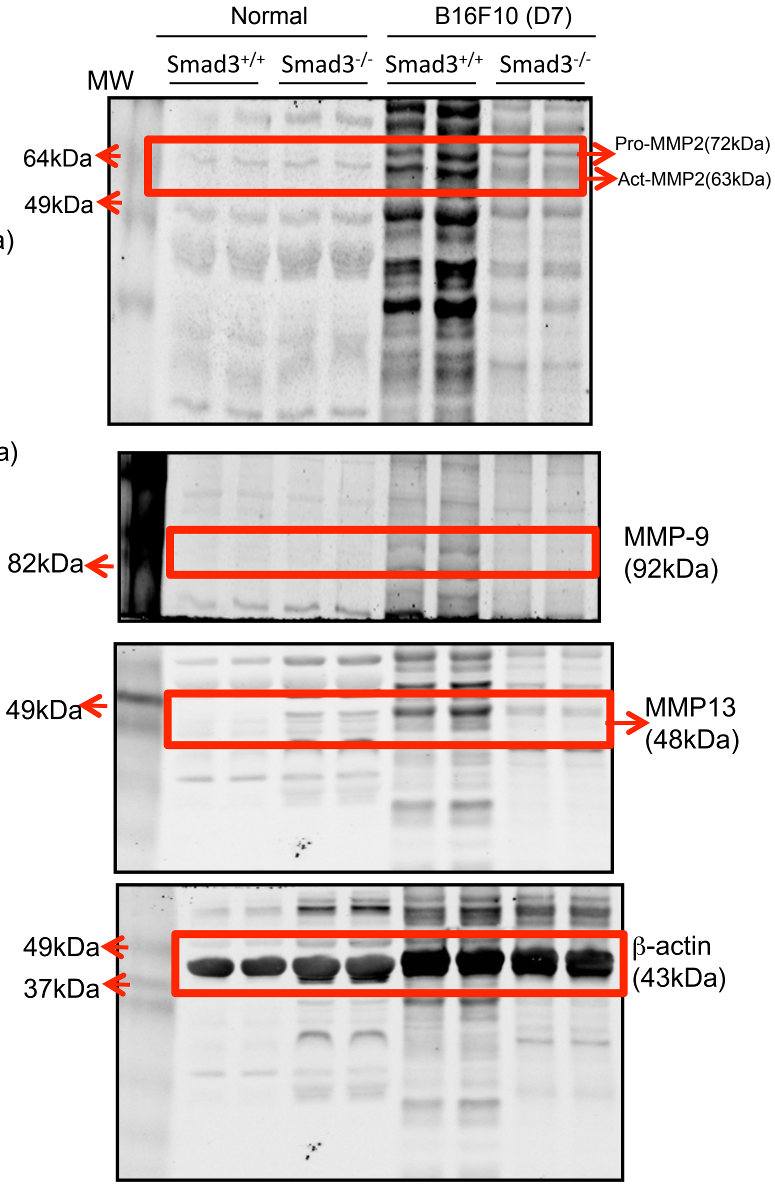

Supplementary Figure 5E

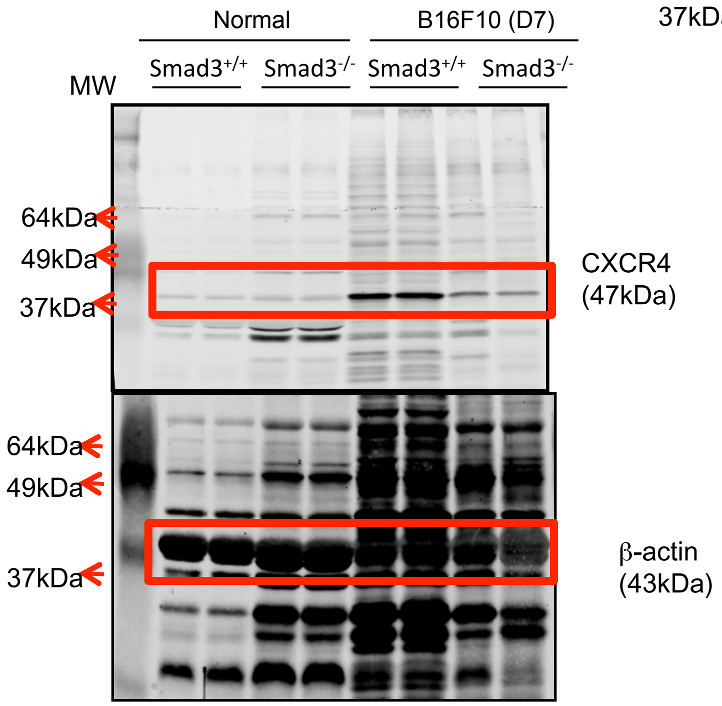

Supplementary Figure 18. Full-size scans of Western blots in Supplementary Figure 5.

Supplementary Figure 12A

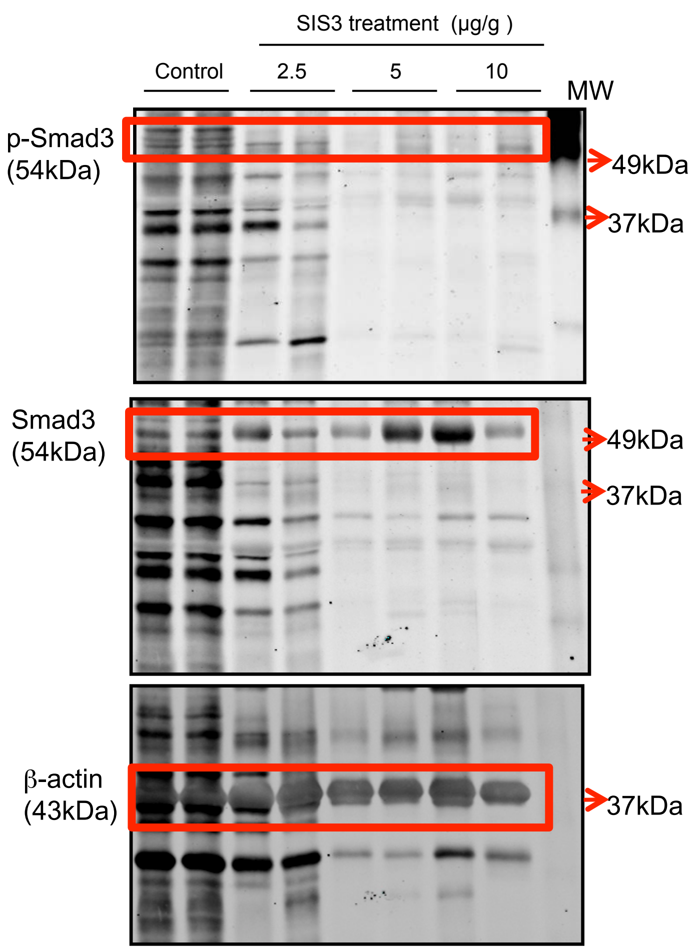

Supplementary Figure 12B

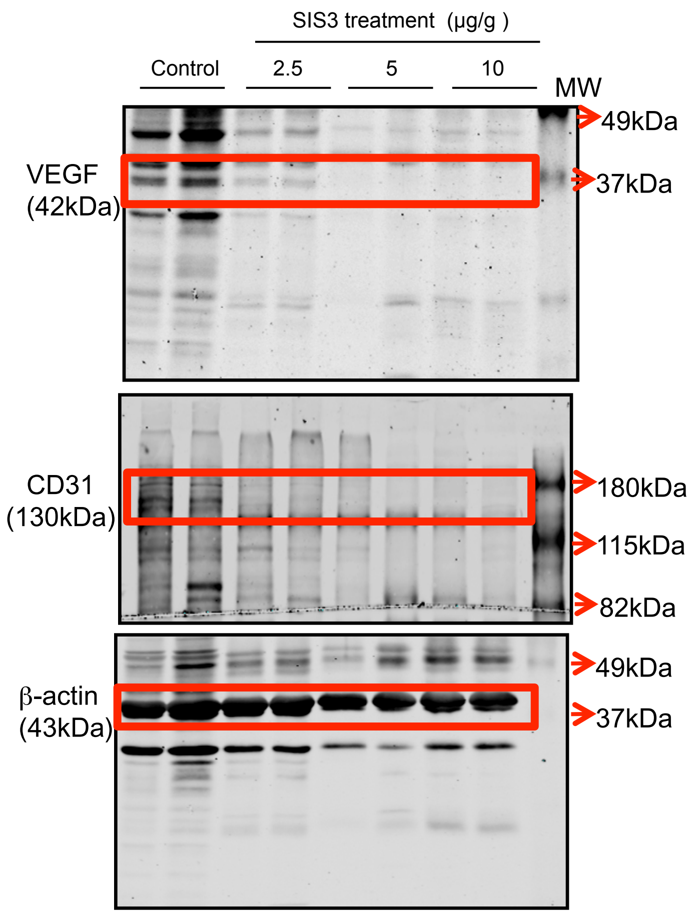

Supplementary Figure 19. Full-size scans of Western blots in Supplementary Figure 12.

Supplementary Figure 12C

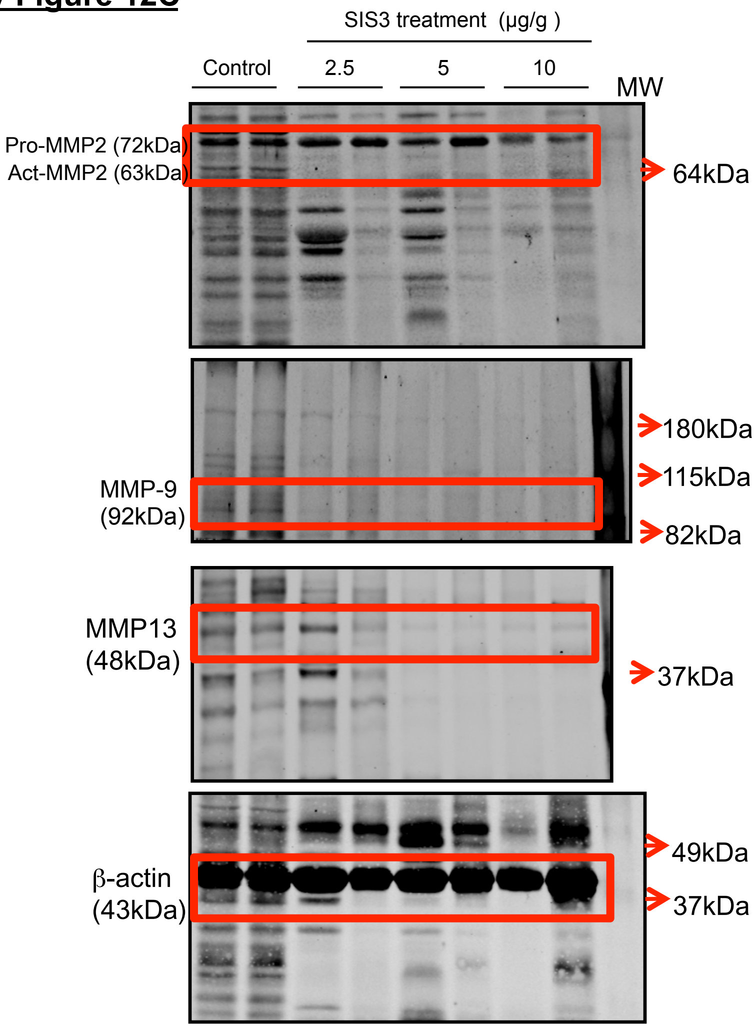

Supplementary Figure 12D

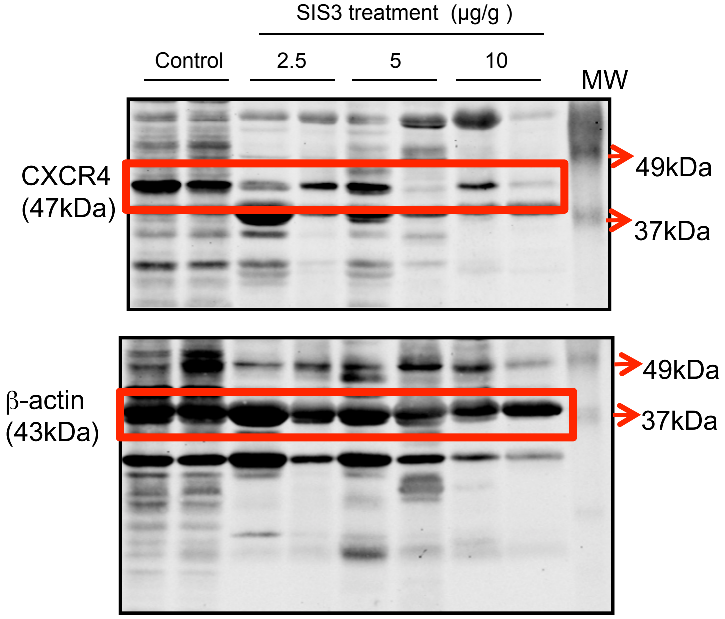

Supplementary Figure 20. Full-size scans of Western blots in Supplementary Figure 12.
